# Gene Expression in the Skin of Dogs Sensitized to the House Dust Mite *Dermatophagoides farinae*

**DOI:** 10.1534/g3.114.013003

**Published:** 2014-08-05

**Authors:** Paz Schamber, Rachel Schwab-Richards, Stefan Bauersachs, Ralf S. Mueller

**Affiliations:** * Centre for Clinical Veterinary Medicine, Ludwig-Maximilians-Universität München, 80539 Munich, Germany; † Novartis Centre de Recherche, 1566 Saint-Aubin, Switzerland; ‡ Laboratory for Functional Genome Analysis, Ludwig-Maximilians-Universität München, 81377 Munich, Germany

**Keywords:** *Canis familiaris*, canine, Beagle, allergy, microarray

## Abstract

Atopic dermatitis is a multifactorial allergic skin disease in humans and dogs. Genetic predisposition, immunologic hyperreactivity, a defective skin barrier, and environmental factors play a role in its pathogenesis. The aim of this study was to analyze gene expression in the skin of dogs sensitized to house dust mite antigens. Skin biopsy samples were collected from six sensitized and six nonsensitized Beagle dogs before and 6 hr and 24 hr after challenge using skin patches with allergen or saline as a negative control. Transcriptome analysis was performed by the use of DNA microarrays and expression of selected genes was validated by quantitative real-time RT-PCR. Expression data were compared between groups (unpaired design). After 24 hr, 597 differentially expressed genes were detected, 361 with higher and 226 with lower mRNA concentrations in allergen-treated skin of sensitized dogs compared with their saline-treated skin and compared with the control specimens. Functional annotation clustering and pathway- and co-citation analysis showed that the genes with increased expression were involved in inflammation, wound healing, and immune response. In contrast, genes with decreased expression in sensitized dogs were associated with differentiation and barrier function of the skin. Because the sensitized dogs did not show differences in the untreated skin compared with controls, inflammation after allergen patch test probably led to a decrease in the expression of genes important for barrier formation. Our results further confirm the similar pathophysiology of human and canine atopic dermatitis and revealed genes previously not known to be involved in canine atopic dermatitis.

Atopic dermatitis (AD) is a chronic inflammatory skin disease with an increasing prevalence in both humans and dogs in industrialized countries (Leung 1999; Hillier and Griffin 2001). It manifests as a recurrent, pruritic dermatitis, in most cases with allergen-specific serum-IgE (Wüthrich 1978; Lian and Halliwell 1998; Olivry *et al.* 2001; Halliwell 2006), and is often complicated by secondary skin infections (Leyden *et al.* 1974; Deboer and Marsella 2001). Diagnosis of canine AD (cAD) is based on history, clinical signs, and the exclusion of other causes of pruritus (Deboer and Hillier 2001). Spontaneous remission of cAD is rare and therapy can be unsatisfying (Olivry and Sousa 2001; Olivry *et al.* 2010). Allergen-specific immunotherapy, the only specific treatment for the disease, is a long-term therapy and is not efficacious in all patients (Olivry *et al.* 2010). In many patients, lifelong symptomatic therapy is needed.

The pathophysiology of cAD is still not fully elucidated. A complex interaction between environmental and genetic factors seems to affect skin barrier function and the immunologic response of patients in both human and canine AD (Marsella *et al.* 2011). In linkage analyses and candidate gene studies, associations to and polymorphisms in different epidermal and immunologic genes were identified in AD (Cookson and Moffatt 2002; Barnes 2010; Boguniewicz and Leung 2011; Bussmann *et al.* 2011). A few years ago a central role of the barrier abnormality in atopic skin was proposed for AD (the outside–inside view of disease pathogenesis) (Taieb 1999; Elias and Feingold 2001). In human AD, a defective skin barrier is hypothesized to facilitate the penetration of allergens into the skin, which leads to sensitization against environmental allergens and subsequent cutaneous inflammation, which further aggravates skin barrier impairment. The release of the canine genome sequence in 2005 (Lindblad-Toh *et al.* 2005) permitted functional genome analysis and the analyses of gene expression patterns in the dog. With microarray technology, expression of thousands of genes can be evaluated simultaneously to identify differentially expressed genes (DEG) as candidate genes for further studies (Schulze and Downward 2001).

The aim of this study was to compare the gene expression pattern before and after challenge with an allergen patch test in the skin of house dust mite–sensitized dogs compared with nonsensitized controls in a highly standardized manner to identify new genes possibly involved in the pathophysiology of AD.

## Materials and Methods

Microarray (MA) technique is a sensitive method for the measurement of gene expression. To avoid data corruption and/or misinterpretation due to environmental conditions or aberrant/differing age of skin lesions, conditions for the biological model, specimen collection, and preparation were highly standardized.

### Animals and specimen collection

A total of 12 Beagle dogs owned by the Novartis Centre de Recherche Sante Animale (Saint-Aubin FR, Switzerland) were included in the study. Six of these dogs were previously sensitized to *Dermatophagoides farinae* (*D.f.*) (four males and two females) and six were nonallergic control dogs (two males and four females). The former dogs had been sensitized epicutaneously once weekly for 8 wk to the house dust mite *Dermatophagoides farinae* (powder of milled dust mite, 99% pure *Dermatophagoides farinae*, mixed with saline to a pasty consistency; Greer Laboratories, North Carolina). Successful sensitization was confirmed with two challenges using a D.f. slurry; all dogs had to develop skin lesions over a predefined score. Subsequent studies showed that they also had serum D.f.-specific IgE and positive intradermal tests for D.f. The age of the dogs ranged from 2 to 3 yr (mean, 2.3 yr). Environmental and feeding conditions were the same for all dogs. This study was approved by the appropriate regulatory office.

A 10- × 10-cm area on the lateral chest of each dog was shaved with a clipper blade (Favorita II GT 104; Aesculap AG, Germany). Four days later, a patch test (PT) was conducted with 75 mg of HDM paste (powder of milled dust mite, 99% pure *Dermatophagoides farinae*, mixed with saline to a pasty consistency; Greer Laboratories, North Carolina) and 50 μl of physiologic saline as a negative control. The patch test sites were bandaged carefully to prevent trauma or movement of the patches. In addition, each dog wore a mesh body suit. The allergen patch test was carefully relocated in the exact same position and bandages were replaced at the 6 hr intervention. In total, five 8-mm punch biopsies were performed in each dog using local anesthesia; each site used 0.5 ml of 2% lidocaine (Vetoquinol AG, Ittingen, Switzerland). One specimen was obtained before (0 hr) patch test and two biopsy specimens were obtained at 6 hr and at 24 hr (6 hr, 24 hr) after patch test with both allergen (A) and saline (S) applications (Supporting Information, Table S1 in File S1). After clipping and at each time point (0 hr, 6 hr, 24 hr) before collecting the biopsy samples, skin was evaluated for signs of inflammation. To investigate the cellular response and gene expression in the skin, biopsy specimens were cut into three pieces with two parallel cuts. The middle part of each specimen was placed in 10% neutrally buffered formalin. The other two pieces were placed into RNAlater (Qiagen GmbH, Hilden, Germany) and after 4 d of storage at 4°, these specimens were stored at −20° until further processing.

### Histological evaluation

For quantitative histological analysis, formalin-fixed specimens were embedded in paraffin and routinely stained with hematoxylin and eosin (HE). Specimens were stained by the Institute of Veterinary Pathology in Munich. Sections were then photographed (ColorView III; Olympus Soft Imaging Solutions GmbH) and round cells were counted (cells/mm^2^) with analySIS FIVE Software (Soft Imaging System; Olympus).

### Microarray analysis

Total RNA was isolated from the skin specimen using Trizol reagent (Life Technologies GmbH, Darmstadt, Germany) according to the manufacturer’s instructions. The quantity and purity of RNA were measured with a NanoDrop 1000 (Peqlab Biotechnologie GmbH, Erlangen, Germany). The quality of total RNA was determined electrophoretically with an Agilent 2100 Bioanalyzer (RNA 6000 Nano Kit, 5067-1511; Agilent Technologies, Waldbronn, Germany). Transcriptome analysis was performed with an Agilent 8x60K Canine Custom Gene Expression Array. The canine custom array was designed using the Agilent webtool "eArray." Relevant genes based on previous studies of human and canine AD were included in the array design. Cy3-labeled cRNA was produced with the low-input Quick Amp Labeling Kit one color (Agilent Technologies) and after fragmentation hybridized to the MAs according the manufacturer’s instructions. Hybridized and washed slides were scanned at 2-µm resolution with a DNA Microarray Scanner G2505C (Agilent Technologies). Image processing was performed with Feature Extraction Software 10.7.3.1 (Agilent Technologies). Processed signals were filtered based on "is well above background" flags (detection in at least five of six samples in at least one group). Thereafter, expression data were normalized using the Bioconductor package "vsn" (Huber *et al.* 2002) in "R" (version 2.12.2; http://www.r-project.org/). For quality control, microarray data were analyzed with box plots before and after normalization and a heatmap based on pair-wise distances. Significance analysis was performed with the package "limma" (version 3.6.9) (Smyth 2005). Significance thresholds were set at a false discovery rate (FDR) of 5% and fold change (FC) of at least 1.5-fold.

To characterize gene expression responses in the skin of sensitized dogs after allergen PT, an unpaired two-class analysis was performed for the 0-hr biopsy specimens to find general differences in gene expression between sensitized and nonallergic dogs in untreated skin. Furthermore, an unpaired multifactorial analysis was performed for the 6 hr and the 24 hr specimens comparing the differences of allergen with saline application between allergic and control dogs.

The array annotation was complemented based on Ensembl, Entrez Gene, and BLAST analyses to obtain canine and human (putative orthologous) gene IDs. After statistical analysis, the values of genes represented by more than one microarray probe were summarized using the "Group" tool implemented in the Galaxy software (http://galaxy.psu.edu/).

### Functional analysis of microarray data

Multi Experiment Viewer Software (version 4.7.1; http://www.tm4.org/) (Saeed *et al.* 2003) was used for hierarchical cluster (HCL) and self-organizing tree algorithm (SOTA) analysis. For these analyses, the log2 transformed mean expression value of each group was subtracted from the mean expression value of all groups. Functional classification of differentially expressed genes (DEG) was conducted using the "functional annotation clustering" and the "functional annotation chart" tools of the Database of Annotation, Visualization, and Integrated Discovery (DAVID; http://david.abcc.ncifcrf.gov/home.jsp) (Huang *et al.* 2008), the Kegg Mapper Pathway analysis tool of the Kyoto Encyclopedia of Genes and Genomes (KEGG) database (http://www.genome.jp/kegg/tool/map_pathway1.html) (Kanehisa *et al.* 2008), and the keyword enrichment tool CoPub (http://services.nbic.nl/copub5) (Frijters *et al.* 2008). Analyses were performed based on Entrez Gene IDs of the putative human orthologous genes.

### Quantitative real-time RT-PCR

The results of the MA analysis were verified by validating the expression of selected DEG by quantitative real-time RT-PCR (qPCR). Specific primers were designed using the NCBI Primer-BLAST tool (http://www.ncbi.nlm.nih.gov/tools/primer-blast/) and synthesized by Thermo Fisher Scientific GmbH (http://www.thermoscientific.com/biopolymers; Germany). The sequences of the used qRT-PCR-Primer are shown in Table S2. The same RNA samples were used for qPCR and MA analysis. First-strand cDNA was synthesized starting from 1 µg of total RNA with the Sprint RT Complete-Double PrePrimed 48-well strips Kit (Takara Bio Europe/Clontech, Saint-Germain-en-Laye, France) according to the manufacturer’s instructions. A two-step qPCR experiment was performed (PMID 22031715) (Streyl *et al.* 2012). The Power SYBR Green PCR Master Mix and RT-PCR (Applied Biosystems, Darmstadt, Germany) was applied in the thermal cycler (StepOne Real-Time PCR System; Applied Biosystems) and evaluated with the qPCR StepOne Software (V 2.2.2; Applied Biosystems. The cycle thresholds (CTs) determined for the target genes were normalized against the geometric mean of the reference genes *RLPL13A*, *LOC479750* (*CCZ1*) as described by Wood *et al.* (2008) and *UBB* to obtain ∆CT values. Quantitative PCR results were statistically evaluated with the same parameters as microarray data in limma.

## Results

### Clinical evaluation of patch test sites

The skin of all dogs showed no abnormal dermatologic findings after clipping and at the 0-hr and 6 hr time points for both allergen (n = 6) and saline-treated skin (n = 6). The skin of the control group was also unremarkable after 24 hr. At that time, three of the six sensitized dogs showed mild erythema at the allergen (A) site but no visible reactions at the control (S) site.

### Round cell counts

**Figure 1:**
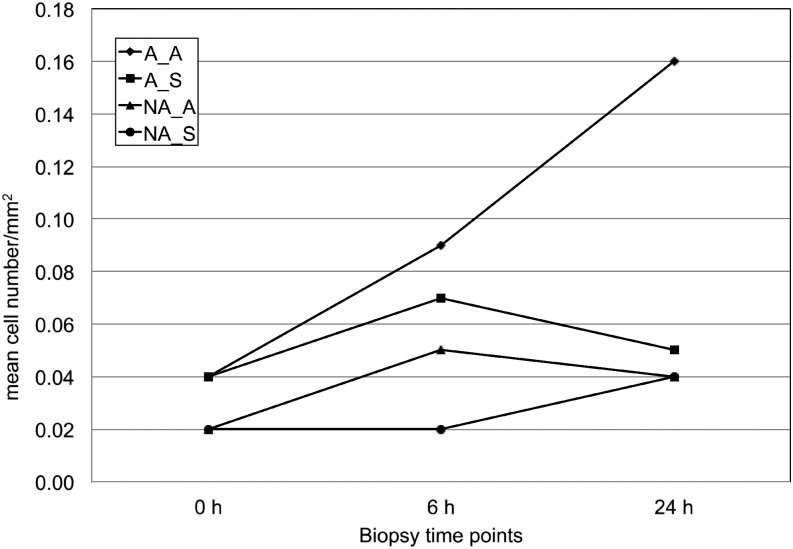
Round cell counts in skin biopsy specimens from patch test sites of allergic and normal dogs before and 6 hr and 24 hr after application of allergen and negative control (saline). A_A = skin of **a**llergic dogs after **a**llergen patch test; A_S = skin of **a**llergic dogs after **s**aline patch test; NA_A = skin of **n**on**a**llergic dogs after **a**llergen patch test; NA_S = skin of **n**on**a**llergic dogs after **s**aline patch test.

Analysis of round cell counts showed increasing round cell numbers in the skin of sensitized dogs treated with allergen (Figure 1). However, the difference was not statistically significant when comparing the skin specimens for all time points and treatments (0 hr, 6 hr saline, 6 hr allergen, 24 hr saline, 24 hr allergen) (Kruskal Wallis test, *P* = 0.49 and *P* = 0.08 in nonsensitized and sensitized dogs, respectively).

### Microarray results

RNA integrity number values (RIN) of the 60 isolated total RNA samples ranged from 7.0 to 8.6, except for one sample (no. 14) that had RIN of 3.3. DNA microarrays representing more than 22,000 unique canine genes were used to compare the global gene expression profiles. After data processing and normalization, the quality control (box plots and pairwise distance heatmap) of the data set revealed lower total signal intensity and poor correlation to all other samples for sample 14 (sensitized dog 24 hr after allergen application). Based on this result and the poor integrity of this sample, the corresponding data set was excluded from further analysis.

Statistical analysis was performed to identify differential gene expression after treatment with allergen and saline control between sensitized and nonsensitized control dogs (Table 1). No significant expression differences were found before treatment (0 hr) between sensitized and healthy control dogs. The unpaired multifactorial analysis of the 6 hr time point showed only nine differentially expressed genes (DEG). Of these, only five could be assigned to hitherto known genes (Table 2). The unpaired multifactorial analysis of the skin biopsy specimens 24 hr after treatment revealed 587 DEG; 361 were upregulated and 226 were downregulated in the skin of sensitized dogs (Table 3). The complete list of DEG between sensitized and nonsensitized dogs 24 hr after patch test with allergen and saline is provided in the File S2.

**Table 1:** Statistical analysis and differentially expressed genes in allergic and nonallergic dogs after patch testing with allergen and saline

Comparison	Groups Included and Calculation	Up	Down
Two-class unpaired, 0 hr	A0hr-N0hr	0	0
Unpaired multifactorial, 6 hr	(A.a0.6hr-A.s0.6hr)-(N.a.6hr-N.s.6hr)	6	3
Unpaired multifactorial, 24 hr	(A.a.24hr-A.s.24hr)-(N.a.24hr-N.s.24hr)	361	226

A, allergic; a, allergen; s, saline; N, nonallergic.

**Table 2:** Differentially expressed genes 6 hr after PT

Cfa*^a^*: Entrez GeneID	Cfa: Gene Symbol	Primary Accession	RefSeq Accession	Genbank Accession	Hsa: Entrez GeneID	Hsa: Gene Symbol	Coeff.	p-value*^a^*	p-value adj.*^b^*
482051	*ZFAT*	ENSCAFT00000001794	XM_843165	ENSCAFT00000001794	482051	*ZFAT*	−0.651	0.0003	0.999
—	—	CO597689		CO597689	—	*—*	−0.725	0.0006	0.999
—	—	BI421087			—	*—*	−0.809	0.0009	0.999
—	—	TC78107			—	*—*	0.972	0.0009	0.999
403981	*CCL2*	NM_001003297	NM_001003297	NM_001003297	—	*—*	1.973	0.0015	0.999
448792	*CCL8*	NM_001005255	NM_001005255	NM_001005255	448792	*CCL8*	1.953	0.0017	0.999
—	—				—	*—*	0.632	0.0006	0.999
485412	*DBX1*			XM_542531	485412	*LOC486388*	0.697	0.0002	0.999
490156	*GFI1*	NM_001012719	NM_001012719	NM_001012719	490156	*GFI1*	0.827	0.0008	0.999

Cfa, Canis lupus familiaris; Hsa, Homo sapiens; Coeff., log2 fold change.

a p-value = nominal p-value.

b p-value adj. = p-value corrected for multiple testing.

**Table 3:** Gene set 1 and set 2 results of DAVID analysis with the highest enrichment scores

Gene Set	Functional Annotation Cluster Description*^a^*	Enrichm. Score*^b^*	Genes*^c^*
1	Immune response (49; 4.73)*^d^*; inflammatory response (31; 6-36); response to wounding (37; 4.65); defense response (39; 4.23)	15.29	65
2	Epidermis development (13, 8.53); epidermal cell differentiation (7; 11.74); epithelial cell differentiation (8; 7.05); keratinocyte differentiation (6, 10.98), epithelium development (9;0.4.79); cornified envelope (3; 16.60); keratinization (3; 8.43)	4.14	14
2	Cytoskeleton (27; 2.38)	3.23	27
2	Cell junction (16; 3.76); anchoring junction (9; 6.37); apical junction complex (6; 7.38); adherens junction (7; 5.50)	2.22	16
2	Desmosome (3; 18.26), tight junction (3; 5.84)	1.58	6

a Based on the most prominent "terms."

b Geometric mean (in −log10) of p-values of groups with corresponding "annotation clusters."

c Total number of genes in one cluster.

d In parentheses: number of genes and the "fold enrichment of the functional term."

### Bioinformatics analysis of DEG 24 hr after PT

To visualize gene expression changes between treatments and sensitized compared with control dogs, hierarchical cluster analysis (HCL) and clustering of similar expression profiles over time [self-organizing tree algorithm (SOTA) analysis] were performed based on expression values relative to the mean expression value of all samples for each gene, respectively. The input for the SOTA analysis was: Metric: Pearson correlation, max. cycles: 3, with default parameters [mean-centered log2-transformed normalized expression values (vsn value − mean of vsn values of all samples)]. HCL analysis was performed with the DEG of the 24 hr unpaired multifactorial analysis to group these genes based on similarity in expression across the treatment groups and to cluster the groups on the basis of similarities in gene expression pattern (Figure 2). Basically, this analysis revealed two major gene sets. Each gene set was then used to extract functional gene sets by “functional annotation clustering” and “functional annotation chart” analyses with DAVID. DAVID analysis of gene set 1, which contained genes with increased expression in sensitized dogs, revealed genes related to "immune response" and "inflammatory response" (Table 3). In contrast, genes belonging to gene set 2 (decreased in sensitized dogs) were known to be involved in epidermal development and skin barrier formation (Table 3). Detailed results of DAVID analyses of gene set 1 and gene set 2 and the used gene lists are provided in File S3.

To determine if there were gene sets with a similar expression profile over time, SOTA analysis was performed and genes were assigned to four clusters (Figure 3). Genes of the first cluster showed increased expression in both sensitized and nonsensitized dogs after saline and allergen treatment. The allergen-treated skin of sensitized dogs showed the greatest increase. There was no further increase between the 6 hr and the 24 hr time points in the allergen-treated skin of nonsensitized dogs. Genes of cluster 1 were associated with functional categories such as "immune response," "inflammatory response," and "response to injury." Genes of the second cluster showed a reverse expression profile to the first cluster with decreasing expression over time. The strongest decrease was seen in the allergen-treated skin of sensitized dogs. There was no further decrease, but rather an increase between 6 hr and 24 hr in allergen-treated skin of nonsensitized dogs. For the genes of cluster 2, the functional terms "epidermis development," "epidermal differentiation," and "keratinocyte differentiation" showed the highest enrichment scores. *Prima facie* cluster 3 and cluster 4 showed equal expression behavior. In both gene clusters, the expression in the skin of allergen-treated sensitized dogs decreased between 6 hr and 24 hr. The expression in the other groups increased to that of the 6 hr biopsy and then also decreased to that of the 24 hr time point, with one exception in cluster 3, where the expression increased between the 6 hr and 24 hr time points in the skin of allergen-treated nonsensitized dogs. Cluster 3 and cluster 4 contained both genes related to cell junctions. Details of the DAVID results for the SOTA clusters are shown in File S4.

DEG were further categorized and genes of interest were selected based on a literature review and the literature proposed by CoPub (Table 4).

**Table 4:** Selected DEG of interest with corresponding fold changes (FC)

Human Gene Symbol	Human Entrez Gene ID	Cfa Gene Symbol	Cfa Entrez/ Ensembl Gene ID	Human Gene Description	Coeff. Diff_AvsN_24h	FC	p-value	p-value adj.
*CCL8*	6355	*CCL8*	448792	Chemokine (C-C motif) ligand 8	2.07	4.2	0.0009	0.0483
*FCGR3A*	2214	*FCGR3A*	478984	Fc fragment of IgG low affinity IIIa receptor (CD16a)	0.96	1.9	0.0000	0.0116
*IL13RA2*	3598	*IL13RA2*	403622	Interleukin 13 receptor alpha 2	2.15	4.4	0.0000	0.0014
*IL18BP*	10068	*IL18BP*	476818	Interleukin 18 binding protein	2.27	4.8	0.0001	0.0160
*IL33*	90865	*IL33*	403810	Interleukin 33	1.73	3.3	0.0000	0.0143
*SOCS3*	9021	*SOCS3*	442949	Suppressor of cytokine signaling 3	1.32	2.5	0.0008	0.0524
*CHI3L1*	1116	*CHI3L1*	490222	Chitinase 3-like 1 (cartilage glycoprotein-39)	2.18	4.5	0.0009	0.0550
*CLEC7A*	64581	*CLEC7A*	611385	C-type lectin domain family 7 member A	0.64	1.6	0.0011	0.0591
*BCL3*	602			B-cell CLL/lymphoma 3	0.72	1.7	0.0010	0.0565
*CXCR6*	10663	*CXCR6*	608840	Chemokine (C-X-C motif) receptor 6	0.68	1.6	0.0003	0.0312
*ADORA2B*	136	*ADORA2B*	403410	Adenosine A2b receptor	0.71	1.6	0.0017	0.0740
*FCGR2B*	2213			Fc fragment of IgG low affinity IIb receptor (CD32)	1.36	2.6	0.0005	0.0385
*MRC1*	4360	*LOC487114*	487114	Mannose receptor C type 1	1.60	3.0	1.0E−05	0.0072
*OSMR*	9180	*OSMR*	489223	Oncostatin M receptor	1.03	2.0	0.0001	0.0160
*TNFAIP6*	7130	*TNFAIP6*	476147	Tumor necrosis factor alpha-induced protein 6	2.61	6.1	0.0013	0.0649
*TNFSF9*	8744	*TNFSF9*	476729	Tumor necrosis factor (ligand) superfamily member 9	1.32	2.5	0.0003	0.0320
*TNFSF13B*	603969	*TNFSF13B*	485545	Tumor necrosis factor (ligand) superfamily member 13b	1.07	2.1	0.0005	0.0353
*TGM1*	7051	*TGM1*	403630	Transglutaminase 1 (K polypeptide epidermal type I protein-glutamine-gamma-glutamyltransferase)	−0.60	−1.5	0.0002	0.0283
*DSP*	1832	*DSP*	488207	Desmoplakin	−0.63	−1.5	0.0004	0.0357
*SPINK5*	11005	*SPINK5*	478055	Serine peptidase inhibitor Kazal type 5	−0.83	−1.8	0.0011	0.0601
*FLG2*	388698		ENSCAFT00000020610	Filaggrin family member 2	−1.88	−3.7	0.0000	0.0150
*CALML5*	51806	*CALML5*	487146	Calmodulin-like 5	−0.73	−1.7	0.0019	0.0761
*ASPRV1*	151516	*ASPRV1*	481416	Aspartic peptidase retroviral-like 1	−1.25	−2.4	0.0000	0.0114
*OCLN*	4950	*OCLN*	403844	Occludin	−0.66	−1.6	0.0001	0.0176
*PPARA*	5465			Peroxisome proliferator-activated receptor alpha	−0.84	−1.8	0.0005	0.0397
*PPL*	5493	*PPL*	490021	Periplakin	−0.64	−1.6	0.0003	0.0339
*SCEL*	8796			Sciellin	−1.18	−2.3	0.0001	0.0226
*GATA3*	2625	*GATA3*	487134	GATA binding protein 3	−0.84	−1.8	0.0012	0.0620
*LOR*	4014	*LOR*	609440	Loricrin	−1.40	−2.6	0.0011	0.0591
*DMKN*	93099	*DMKN*	476484	Dermokin	−0.92	−1.9	0.0004	0.0359
*KPRP*	448834	*KPRP*	ENSCAFG00000013003	Keratinocyte proline-rich protein	−1.42	−2.7	0.0006	0.0443
*SGMS1*	259230	*LOC477583*	ENSCAFT00000031827	Sphingomyelin synthase 1	−0.59	−1.5	0.0007	0.0468
*ALOXE3*	59344	*ALOXE3*	489487	Arachidonate lipoxygenase 3	−0.64	−1.6	0.0015	0.0697
*DSG1*	1828	*DSG1*	403401	Desmoglein 1	−0.68	−1.6	0.0008	0.0519
*ASAH2*	56624	*ASAH2*	486461	N-acylsphingosine amidohydrolase (nonlysosomal ceramidase) 2	−0.93	−1.9	0.0008	0.0506
*CGN*	57530	*CGN*	483198	Cingulin	−0.98	−2.0	0.0001	0.0158

### Validation of microarray results by quantitative real-time RT-PCR

To validate MA results, 18 of these genes were selected for quantitative PCR (qPCR). Results were analyzed with limma similar to the MA data. Overall, expression differences identified by MA analysis were confirmed (Table 5). For some of the analyzed genes, p-values were not significant (p>0.05) because of variations in expression differences between dogs.

**Table 5:** Comparison of unpaired multifactorial results between microarray and qPCR data

		Microarray				qPCR			
Up	Gene Symbol	Coeff.	FC	p-value	p-value adj.	Coeff. C_T_ value	FC	p-value	p-value adj.
	*CCL8*	2.07	4.2	0.0009	0.0483	−3.68	12.8	0.0002	0.0005
	*FCGR3A*	0.96	1.9	0.0000	0.0116	−2.02	4.1	0.0130	0.0207
	*IL13RA2*	2.15	4.4	0.0000	0.0014	−2.44	5.4	0.0000	0.0000
	*IL18BP*	2.27	4.8	0.0001	0.0160	−3.24	9.4	0.0002	0.0004
	*IL33*	1.73	3.3	0.0000	0.0143	−2.08	4.2	0.0001	0.0004
	*SOCS3*	1.32	2.5	0.0008	0.0524	−1.80	3.5	0.0614	0.0701
	*CLEC7A*	0.64	1.6	0.0011	0.0591	−1.17	2.2	0.0272	0.0680
Down									
	*TGM1*	−0.60	−1.5	0.0002	0.0283	0.50	−1.4	0.0481	0.0642
	*DSP*	−0.63	−1.5	0.0004	0.0357	0.57	−1.5	0.1045	0.1045
	*FLG2*	−1.41	−2.7	0.0001	0.0166	1.65	−3.1	0.0227	0.0680
	*DMKN*	−0.92	−1.9	0.0004	0.0359	1.06	−2.1	0.0466	0.0777
	*ALOXE3*	−0.64	−1.6	0.0015	0.0697	0.61	−1.5	0.1404	0.1404
	*DSG1*	−0.68	−1.6	0.0008	0.0519	0.93	−1.9	0.0627	0.0783
	*SPINK5*	0.83	−1.8	0.0011	0.0601	0.76	−1.7	0.0393	0.0514
	*OCLN*	−0.66	−1.6	0.0001	0.0176	0.72	−1.6	0.0000	0.0002
	*PPARA*	−0.84	−1.8	0.0005	0.0397	1.01	−2.0	0.0014	0.0023
	*LOC476953/SCEL*	−1.78	−2.3	0.0001	0.0226	1.38	−2.6	0.0001	0.0002
	*KPRP*	−1.42	−2.7	0.0006	0.0443	1.79	−3.5	0.0003	0.0008

## Discussion

This study evaluated the cutaneous gene expression of six normal and six house dust mite–sensitized dogs under controlled environmental conditions using patch tests. Two patches were applied to each dog, one with the allergen (D.f.) and the other with saline. Biopsy specimens were collected before and at 6 hr and 24 hr after patch application. We identified 587 differentially expressed genes between sensitized and normal control dogs. In our setting, variables such as breed, age, chronicity, and living conditions were standardized. In previous studies, dogs with naturally occurring disease were evaluated (Merryman-Simpson *et al.* 2008; Wood *et al.* 2009) and their variation in the above listed factors made it more difficult to identify subgroups of genes deregulated in AD. As expected, no dog showed dermatologic changes after saline treatment, similar to the findings of Marsella *et al.* (2006). The clinical change detected after 24 hr is in agreement with our histological findings, in which the influx of inflammatory cells was limited to the allergen-treated skin of the sensitized dogs.

Microarray analysis of skin biopsy samples collected before the patch test (0 hr) did not reveal any DEG. This supports the hypothesis that artificially sensitized dogs should not suffer from a genetically determined defective skin barrier and corresponding gene expression changes. Furthermore, these results suggest that sensitization does not induce a permanent expression change of genes involved in inflammation, but rather an ability for disturbed reactivity on re-exposure to allergen.

Unexpectedly, the multifactorial analysis of the 6 hr biopsy specimens revealed only nine DEG, and of these genes only five were sufficiently annotated. Because only a few genes were upregulated or downregulated even before the adjustment, it seems likely that the short time period did not permit changes of sufficient magnitude to reach statistical significance. The inflammatory chemokine (C-C motif) ligands (CCL) 2 and 8 showed the highest gene expression changes.

In contrast, the 24 hr specimen analysis showed 587 DEG. Validation of 18 selected genes by qPCR showed good agreement with the microarray data for these genes. Subsequent bioinformatics analysis of DEG 24 hr after PT assigned these genes to functional groups that are important for skin barrier formation and for an inflammatory response.

In contrast to normal dogs, genes related to inflammation, immune response, and response to injury increased in the skin of sensitized dogs. This was observed to a lesser degree in the control saline patch test area and more prominently with allergen PT. These results could be due to the resultant systemic effects of local allergen exposure, as presumed in human AD (Togias 2004). Most of the genes of the SOTA clusters 2–4 belonging to functional groups important for the skin barrier (epidermal differentiation and cell junctions) showed a reverse expression profile to genes of cluster 1 with a trend to decreased expression. Sensitized dogs again had the strongest changes within the allergen-treated skin. In 2009 Elias and Schmuth postulated their new “outside-inside-outside” hypothesis referring to the pathogenesis of human AD (Elias and Schmuth 2009). They proposed that inherited skin barrier defects lead to increased penetration of allergens and that the subsequent inflammation further aggravates the skin barrier disruption by disturbing the protein synthesis in the stratum corneum. In our dogs, the skin barrier defects seem to be a result of inflammation rather than a preexisting genetic defect, which seems logical in these artificially sensitized dogs, and corresponds to a subset of human patients in which an immunologic defect is responsible for development of atopic disease (Oh *et al.* 2009; Trzeciak *et al.* 2010; Minegishi and Saito 2011). The expression of skin barrier genes in nonsensitized dogs slightly increased between 6 hr and 24 hr. This may be due to regulatory mechanisms that strengthen the skin barrier after an insult.

### DEG related to inflammation

The search for quantitatively enriched functional terms (DAVID) and biological key words (CoPub) associated with the 24 hr DEG revealed the highly enriched functional terms related to inflammation. The immunopathogenesis of AD is characterized by different Th-cell subsets and cytokine profiles. In both atopic dogs and humans, the acute inflammation is characterized by increased T-helper (Th) 2 cells and their cytokines (Leung 1999; Nuttall *et al.* 2002). In chronic inflammation, an increased Th1 response has also been noted in dogs (Nuttall *et al.* 2002).

The genes related to inflammation that were upregulated in sensitized dogs after allergen challenge were chemokines, cytokines, their ligands, receptors involved in the innate and adaptive immune response, costimulatory molecules, and endogenous signaling or transcriptional factors. A number of chemokines [*CCL2*, *CCL3*, *CLL4*, *CCL8*, *CCL13*, *CCL19*, chemokine (C-X-C motif) ligand (CXCL) 1, *CXCL6*, *CXCL16*] were upregulated in sensitized dogs after allergen challenge, similar to what is observed in human atopic dermatitis (Taha *et al.* 2000; Giustizieri *et al.* 2001; Kaburagi *et al.* 2001). Similarly, the cytokine IL-33 was upregulated in our study and was also shown to play a role in human atopic dermatitis and anaphylaxis (Prefontaine *et al.* 2009). More details regarding the genes related to inflammation are discussed in the File S1.

### DEG related to skin barrier function

There is increasing evidence that a defective skin barrier plays an essential role in AD in humans and dogs, although it is not clear in dogs if this defect is primary or secondary. In some humans with AD, genetic mutations lead to a defective skin barrier function and (in combination with environmental factors) to an increased penetration of allergens, resulting in sensitization and development of atopic disease (Elias and Schmuth 2009). Defects of the skin barrier have also been reported in cAD (Inman *et al.* 2001; Hightower *et al.* 2008; Marsella *et al.* 2008). The epidermis serves as a barrier between the body and the environment. Its outermost layer, the stratum corneum, is multilayered and composed of flattened, non-nucleated corneocytes surrounded by a cornified envelope (CE) and multiple planar lamellar sheets and is enriched in ceramides, cholesterol, and free fatty acids (Elias 2005). Normal development of the CE plays a central role for the functions of the stratum corneum (Credille *et al.* 2009). For the physiological properties of the skin, a balance between normal cell proliferation and differentiation and controlled cell desquamation by corneosome degradation are essential (Chapman and Walsh 1990). Filaggrin proteins are essential for the formation of a cornified envelope, and a loss-of-function mutation has been described in humans with atopic dermatitis (Palmer *et al.* 2006). In contrast, in our study, not filaggrin, but rather filaggrin2, a protein with a possible overlapping and synergistic role with filaggrin, was decreased in the allergen-exposed skin of sensitized dogs. There is controversial evidence for the role of filaggrin2 in human atopic dermatitis (Wu *et al.* 2009; Broccardo *et al.* 2011; Marenholz *et al.* 2011). In addition, skin-specific aspartic peptidase retroviral-like 1 (ASPRV1), a protein important for posttranslational processing of profilaggrin to filaggrin (Matsui *et al.* 2011), was downregulated in sensitized dogs after allergen treatment, in contrast to control dogs. Precursors for proteins important for the cornified envelope and enzymes important for crosslinking of such proteins were both downregulated in our sensitized dogs, further implying a role for a postinflammatory change of the epidermal barrier. For the mechanical stability of the skin, intercellular connections such as desmosomes are important. A number of molecules relevant for intercellular adhesions, such as desmoplakin (DSP), desmoglein 1 (DSG1), and tight junction proteins such as occludin (OCLN) participate in the keratinocyte adhesion (Broccardo *et al.* 2011; Ando-Akatsuka *et al.* 1996). *DSP*, *DSG1*, and *OCLN* expression was reduced in allergen PT skin of sensitized dogs. Proteases are involved in the process of corneocyte desquamation (Horikoshi *et al.* 1999) and activate or inactivate antimicrobial peptides such as cathelicidines in the skin (Yamasaki *et al.* 2006). Genes encoding for protease inhibitors such as the gene "serine peptidase inhibitor, Kazal type 5" (*SPINK5*) are important to prevent excessive protease activity resulting in skin barrier defects (Hansson *et al.* 2002; Denecker *et al.* 2008), and polymorphisms in this gene have been shown to be associated with human AD (Walley *et al.* 2001; Nishio *et al.* 2003; Weidinger *et al.* 2008). The sensitized dogs in our study showed reduced expression of SPINK5 24 hr after allergen PT. All those changes point to a defect barrier as a consequence of allergic inflammation in the dogs studied. More details regarding the genes related to the epidermal barrier function are discussed in File S1.

## Conclusion

Our results show that these sensitized dogs developed changes in gene expression related to a defective skin barrier after allergen PT similar to those reported in humans and dogs suffering from naturally occurring AD. Skin inflammation induced by the allergen patch test after 24 hr resulted in changes at the molecular level, which can lead to impaired keratinocyte differentiation and an abnormal development of the cornified envelope. Additionally, the skin barrier was disrupted due to mechanical trauma (scratching). Mechanisms leading to a decreased expression of genes essential for skin barrier formation are largely unknown. In many cases of cAD, clinical signs are probably associated with a combination of several differentially expressed genes rather than one single gene defect. The reduced expression of genes associated with inflammation observed in the control dogs could be due to a negative feedback mechanism, which the sensitized dogs lack. Consequently, in our sensitized dogs, the allergen-induced cutaneous inflammation leads to skin barrier dysfunctions that were induced by sensitization. Furthermore, new genes potentially involved in the pathophysiology of cAD and possible new targets for therapeutic interventions in cAD were identified.

## Supplementary Material

Supporting Information
