# Supplementary material for: Gene Expression in the Skin of Dogs Sensitized to the House Dust Mite Dermatophagoides farinae
Source: G3 (Bethesda). 2014 Aug 5;4(10):1787–95. doi: 10.1534/g3.114.013003 (PMC4199687; doi:10.1534/g3.114.013003)
Supplement: Supporting Information [file supp_4_10_1787__index.html]

Gene Expression in the Skin of Dogs Sensitized to the House Dust Mite Dermatophagoides farinae — Supporting Information 

# Gene Expression in the Skin of Dogs Sensitized to the House Dust Mite *Dermatophagoides farinae*

## Supporting Information for Schamber *et al.*, 2014

**Files in this Data Supplement:**

- Supporting Information - Files S1-S4 and Tables S1-S2 (PDF, 272 KB)
- Table S1 - Design of specimen collection. (PDF, 118 KB)
- Table S2 - Sequences of the oligonucleotides used as primers for qPCR. (PDF, 117 KB)
- File S1 - Supporting Discussion (PDF, 236 KB)
- File S2 - Excel spread sheet showing the data after statistical unpaired analysis. (.xls, 476 KB)
- File S3 - Excel spread sheet with the results of the DAVID analysis of the gene sets 1 and 2; the Annotation Clusters with the highest enrichment score are shown first, the terms give the functional group in wich the genes are classified; in the row GENES the human orthologous gene IDs are listed. (.xls, 460 KB)
- File S4 - The results of the DAVID analysis of genes that were classified in Clusters 1-4 in the SOTA analysis; the annotation clusters with the highes enrichment score are shown first, the row "Term" shows the functional group where the genes were classified in, in the row F "Genes" gene Ids of the genes matching this category are listed. (.xls, 433 KB)
