# Supplementary material for: Gene Expression in the Skin of Dogs Sensitized to the House Dust Mite Dermatophagoides farinae
Source: G3 (Bethesda). 2014 Aug 5;4(10):1787–95. doi: 10.1534/g3.114.013003 (PMC4199687; doi:10.1534/g3.114.013003)
Supplement: Supporting Information [file supp_g3.114.013003_013003SI.pdf]

G3·Genes | Genomes | Genetics

Gene expression in the skin of dogs sensitized to the house dust mite *Dermatophygoides farinae*

Paz Schamber*, Rachel Schwab-Richards§, Stefan Bauersachs†, Ralf S. Mueller*[1]

*Centre for Clinical Veterinary Medicine, Ludwig-Maximilians-Universität München, 80539 Munich, Germany; §Novartis Centre de Recherche, 1566 Saint-Aubin, Switzerland; †Laboratory for Functional Genome Analysis, Ludwig-Maximilians-Universität München, 81377 Munich, Germany

[1]Corresponding author: Centre for Clinical Veterinary Medicine, Ludwig-Maximilians-Universität München, 80539 Munich, Germany, Email: r.mueller@lmu.de

DOI: 10.1534/g3.114.013003

File S1

SUPPORTING DISCUSSION

This study evaluated the cutaneous gene expression of 6 normal and 6 house dust mite sensitized dogs under controlled environmental conditions using patch tests. Two patches were applied to each dog, one with the allergen (*D. farinae*) the other with saline. Biopsies were collected before and at 6 and 24 hours following patch application. We identified 587 differentially expressed genes between sensitized and normal control dogs, but only discussed the biological relevance of those genes very briefly in the manuscript. In this supplemental file we describe selected individual genes and their relevance in more detail.

DEG related to inflammation

Chemokines: All differentially expressed chemokine genes (*CCL2*, *CCL3*, *CLL4*, *CCL8*, *CCL13*, *CCL19*, chemokine (C-X-C motif) ligand (CXCL) 1, *CXCL6*, *CXCL16*) and chemokine receptor 6 (*CXCR6*) were downregulated in the allergen treated skin of non-sensitized dogs, in contrast they were increased in the sensitized dogs. The highest gene expression change was measured for *CCL2* (FC=5). This corresponds to the higher skin and serum concentrations of *CCL2*, *CCL3*, *CCL4* and *CCL13* reported in atopic humans (Taha *et al.* 2000; Giustizieri *et al.* 2001; Kaburagi *et al.* 2001). *CCL2* is known to cause accumulation of Th1 and Th2 cells, monocytes and dendritic cells in humans (Giustizieri *et al.* 2001; Homey *et al.* 2006). *CCL8* has chemotactic properties for highly differentiated CCR8+ Th2 cells in allergic skin and could be jointly responsible for eosinophilic skin inflammation (Debes and Diehl 2011).

The gene expression of interleukin 33 (*IL33*) was decreased in the skin of non-sensitized dogs after allergen treatment, and mRNA concentration was greater in the skin of sensitized dogs. IL33 is a recently described IL1 family member, expressed by different cell types after proinflammatory stimulation (Liew *et al.* 2010). IL33 seems to have an important role in anaphylaxis and atopic dermatitis by promoting IgE-mediated mast cell degranulation (Prefontaine *et al.* 2009). Our results indicate that IL33 may be involved in the inflammation induced by *D.farinae* challenge in this canine model, possibly via influencing IgE-bearing mast cells. If future studies can confirm its role in cAD, it could be a target for new therapeutic interventions in this disease.

Interleukin 13 receptor subunit alpha 2 (*IL13RA2*)*: IL13RA2* mRNA concentration in sensitized dogs was higher in the allergen- than the saline-treated skin. IL13 is a cytokine that plays a pivotal role in activation and maintenance of IgE-production by interacting with the receptor complex of IL13RA1 and IL4RA (Leung *et al.* 2004). Previous studies showed that human AD patients show higher IL13 serum concentration (Katagiri *et al.* 1997) and higher cutaneous IL13 mRNA expression, especially in acute lesions (Hamid *et al.* 1996). IL13RA2 was also increased in the serum of human AD patients (Hussein *et al.* 2011). ILR13A2 binds IL13 with high affinity and it is suspected that this binding of IL13 inhibits its

inflammatory effects (Chomarat and Banchereau 1998). The canine receptor is similar to its human counterpart (Tang 2001) and a similar role in dogs is conceivable. In this study, the normal dogs had lower *IL13RA2* mRNA concentrations in the allergen treated skin. It is possible that because of an absent inflammatory response there was no positive feedback for an increasing *IL13RA2* gene expression. Further studies are needed to elucidate the exact role of this receptor in cAD and its therapeutic potential.

Tumor necrosis factor ligand superfamily member 13B (TNFSF13B): TNFSF13B or "B cell activating factor of the TNF family" (BAFF) is an important survival factor for B cells (Mackay and Schneider 2009). BAFF exists in a membrane-bound and a soluble form (Mackay and Schneider 2009). Increased BAFF serum concentrations were found in people suffering from asthma (Kang *et al.* 2006) and in children with AD (Jee *et al.* 2010). In adults with AD increased BAFF concentrations were found only in acute lesions after allergy patch tests (Chen *et al.* 2011). Thus, BAFF may be more important in early onset, acute lesions of hAD. In our study, gene expression levels of BAFF 24 h after patch test were higher in allergen treated skin of allergic dogs than in control skin, similar to what is seen in humans. In contrast, BAFF levels at the 24 h time point in normal dogs were decreased in allergen treated skin compared to the negative control, indicating BAFF could be involved in a downregulation of inflammation.

TNFSF9: Dendritic cells (DC) are important for antigen (Ag) presentation, T cell stimulation and homing for Ag specific immune response, thus playing a pivotal role in the pathophysiology of AD (Bancherau and Steinmann *et al.* 1998). DC maturation is associated with an increase in the expression of co-stimulatory molecules. One of these cofactors is TNFSF9, a membrane-bound ligand of the TNF family (Wu *et al.* 2011). TNFSF9 binds to its receptor TNFSRF9, which is mainly expressed on activated T and B cells and monocytes (Schwarz *et al.* 1995; Wu *et al.* 2011). Some studies consider TNFSRF9 as a co-receptor for T cell proliferation (Schwarz *et al.* 1995; Wu *et al.* 2011). In contrast, others indicate that activation of this receptor leads to apoptosis of T cells (Langstein *et al.* 1998). Our study showed that in the allergen treated skin, mRNA concentration of *TNFSF9* was decreased in normal dogs but increased in sensitized dogs compared to the negative control. Higher expression of *TNFSF9* in atopic individuals may contribute to the exaggerated immune response. The role of TFNSF9 and its receptor in AD needs to be further investigated.

Suppressor of cytokine signaling proteins (SOCS) 3: The *SOCS3* gene is a member of the SOCS family, a group of transcription factors, induced by cytokines (Elliott and Johnston 2004). SOCS proteins are important for the balance of Th1 and Th2 immune responses (Arakawa *et al.* 2004). *SOCS3* is mainly expressed by Th2 cells. In people suffering from AD, *SOCS3* expression correlates with the severity of clinical signs and further supports Th2 cell differentiation (Seki *et al.* 2003). In cAD a higher expression of SOCS3 was found in lesional and nonlesional skin of allergic dogs compared to non-allergic controls (Schlotter *et al.* 2011). In our study the non-sensitized dogs showed a decrease in *SOCS3* expression 24 h after allergen PT, compared to the other groups. The expression in the control skin was similar to the expression seen in the skin

of sensitized dogs. This result could indicate that non-allergic individuals actively downregulate pro inflammatory pathways which may fail in allergic individuals. If this observation can be confirmed, *SOCS3* may a potential target for AD therapy.

B-cell lymphoma 3-encoded protein (BCL3): *BCL3* was first detected as a proto-oncogene in B cell leukemia (McKeithan *et al.* 1990). *BCL3* is expressed by different cell types such as lymphocytes (Brasier *et al.* 2001) and keratinocytes (Massoumi *et al.* 2006). In keratinocytes *BCL3* expression is stimulated by Th2 cytokines (IL4, IL13), the molecule acts as a transcriptional factor downregulating the expression of genes important for the innate immune response, mainly antimicrobial peptides and upregulating the TNF alpha dependent expression of IL6 and IL8 (Buchau *et al.* 2009). An increase in the BCL3 concentration in lesional skin could be suppressed by vitamin D3 *in vitro* and *in vivo* (Buchau *et al.* 2009). In our study the sensitized dogs showed an increased expression of BCL3 after allergen PT. The non-sensitized dogs showed a prominent decrease of cutaneous BCL3 expression at the site of the allergen patch compared to the saline patch and compared to the expression in sensitized skin at both PT sites. In a similar fashion to the expression of nearly all inflammatory genes evaluated in this study the decrease in the skin of non sensitized dogs at the site of allergen application was prominent, compared to its negative control and to the expression in sensitized skin (at both allergen and saline PT sites). These results suggest that BCL3 may influence secondary skin infections in atopic individuals. Vitamin D3 may reduce the occurrence of such infections by suppressing BCL3 and by increasing cutaneous adenosin monophosphate expression. We can hypothesize that nonallergic individuals actively downregulate exaggerated inflammatory responses.

Adenosine A2B receptor (ADORA) 2B: The endogenous signaling purine molecule adenosine is an important mediator for many different biochemical processes (Berne *et al.* 1983) and has been implicated in playing an increasingly important role in the pathogenesis of asthma (Driver *et al.* 1993). Depending on the binding receptor, adenosine has anti- or pro-inflammatory properties (Rorke and Holgate 2002). In patients suffering from asthma an increased adenosine level was found in the liquid of bronchioalveolar lavage (Driver *et al.* 1993). Adenosine monophosphate causes bronchoconstriction in asthmatic, but not healthy individuals (by interacting with the ADORA2B-adenosine-receptor and mast cell degranulation) (Marquartd and Walker 1990). ADORA2B may have anti- or pro- inflammatory effects or both. Twenty-four hours after allergen PT, sensitized dogs showed an increased ADORA2B mRNA concentration in contrast to normal dogs. To the authors' knowledge a role of adenosine in canine atopic dermatitis has not yet been reported. It is possible that ADORA2B is involved in mast cell degranulation in atopic skin, similar to what has been reported for human asthma. Further studies are needed to investigate if antagonists of ADORA2B could be targets for new therapies in AD.

Fc gamma R: IgE and IgG both may play a role in the pathophysiology of cAD (Willemse *et al.* 1985; Halliwell and DeBoer 2001). IgG may have a protective role and higher IgG serum concentrations have been reported in normal compared to atopic dogs (Lian and Halliwell 1998). In humans patients with extrinsic AD show elevated allergen specific serum IgE and IgG concentrations (Sicherer and Leung 2006). Most investigations on FC-receptors focused on FC*epsilon* receptors because

of the dominant role of IgE in hAD (Kinet 1999). However, IgG can also be increased (DeBoer 1998). Most cells of the immune system have receptors for IgG (FcgammaR). Three different FCgamma receptors, FCgammaI (Cluster of differentiation (CD) 64) FCgammaRII (CD32) and FCgammaRIII (CD16), are known (Ravetch and Kinet 1991). In humans an enhanced expression of CD64 and CD16 was found in acute and chronic atopic skin lesions (Kiekens *et al.* 2000). In an atopic mouse model it was shown that FcepsilonRI (an IgE receptor) and CD16 (an IgG receptor) have overlapping roles (Abboud *et al.* 2009). The normal dogs in our study had a reduced expression of CD32 and CD16 at the allergen PT. Sensitized dogs showed higher mRNA expression of both receptors in the allergen PT skin. Our results suggest that IgG-receptors may be involved in canine atopic skin reactions.

Chitin receptors and chitinases: Chitin is the second most abundant polysaccharide in the environment, after cellulose and it is a component part of different species such as bacteria, mushrooms and insects, but it is not synthesized by mammalians. Recently, it was shown that chitin activates macrophages by interacting with different surface receptors, such as macrophage mannose receptor 1 (MRC1), toll-like receptor 2 (TLR2) (Da Silva *et al.* 2008), dectin 1 (CLEC7A) (Lee 2009), and leukotriene B4 receptor (BLT1) (Reese *et al.* 2007). In our study normal dogs showed a diminished mRNA concentration of MRC1, TLR2 and CLEC7A. In contrast the concentration of these three receptors was increased in the allergen PT skin of sensitized dogs. As chitin is a component of the exoskeleton of house dust mites, these results could indicate possible macrophage activation via chitin-receptor-interaction, contributing to the Th1 activation seen in chronic atopic lesions.

Chitinases are hydrolytic enzymes that are able to degrade chitin. Functional mammalian chitinase genes and chitinase-like proteins (CLP), which are able to bind chitin, but which have lost their enzymatic ability (Renkema *et al.* 1998; Chang *et al.* 2001) have been identified. Recent studies in mammals suggest that both chitinases and CLP are potent regulators of the innate immune response through interaction with chitin molecules (Shibata *et al.* 1997; Lee *et al.* 2008). Some authors suggest an association between CLP and the development and progression of allergic diseases and tissue remodeling (Lee *et al.* 2009; Ober and Chupp 2009). One example for such a CLP is chitinase3-like1 (CHI3L1) coding for its protein YKL40. Its expression is stimulated by IL13 (Lee *et al.* 2009). Increased YKL40 concentrations were found in the serum and lungs of asthma patients and were correlated with the severity of clinical signs. Polymorphisms found in the CHI3L1-gene were correlated with YKL40 concentration and asthma (Chupp *et al.* 2007; Ober and Chupp 2009). Another polymorphism in CHI3L1 has been proposed to be related to atopy in Korean children (Sohn *et al.* 2009). In the present study non sensitized dogs showed a decreased and sensitized dogs an increased CHI3L mRNA concentration in the allergen PT skin. CHI3L1 may play a role in the maintenance of the Th2 inflammation. In previous studies it has been shown that YKL40 prevents the apoptosis of T-cells and macrophages by the inhibition of Fas expression (Lee 2009). Further studies are needed to elucidate the possible role of CHI3L1 and its protein in canine and human atopic dermatitis.

GATA3: GATA transcription factors are a family of transcription factors characterized by their ability to bind to the DNA sequence "GATA". GATA binding protein 3 (GATA3) is a transcription factor inhibiting the Th1- and promoting the Th2-response (Zheng and Flavell 1997; Nawijn *et al.* 2001). In transgenic mice, over-expression of the human GATA3 gene led to an augmented Th2 immune response, which was reversible by inhibiting GATA3 expression (Bae *et al.* 2011). Polymorphisms in GATA3 where shown to be associated with AD in British children (Arshad *et al.* 2008) but not German children (Suttner *et al.* 2009). In contrast to the results in humans the sensitized dogs in this study showed a decreased concentration of GATA3 mRNA. This could be due to the fact that these dogs did not suffer from naturally occurring cAD, but were sensitized. The findings may also be related to this gene pool of dog, if one interprets that the variation between German and British children may be genetically determined but alternatively, GATA3 may not be involved in cAD.

DEG related to skin barrier function

FLG/FLG2: Loss of function mutations and different polymporphisms in the filaggrin (FLG) gene were shown to be associated with hAD (Barker *et al.* 2007; Baurecht *et al.* 2007; Nemoto-Hasebe *et al.* 2009). At this point, there is no evidence that cAD is associated with a defective FLG expression (Chervet *et al.* 2010). A linkage analysis in West Highland White terriers excluded a prominent causative role of canine FLG in the development of atopy in that breed (Barros Roque *et al.* 2009), however Marsella et al. found immunohistochemical changes in FLG in atopic beagles (Marsella *et al.* 2009). Our results revealed neither expression nor change of FLG, but rather a decreased expression of filaggrin2 (FLG2) in allergen treated skin of sensitized dogs at the 24 h patch test. Wu *et al.* identified the FLG2 protein and implied overlapping and perhaps synergistic roles of FLG and FLG2 in the formation of the epidermal barrier (Wu *et al.* 2009). So far, neither a changed gene expression of FLG2 (Wu *et al.* 2009) nor a loss of function mutation in this gene (Marenholz *et al.* 2011) has been detected in hAD. However, a recent study showed a decreased expression of FLG2, desmoglein 1 (DSG1), desmocollin and transglutaminase 3 (TGM3) in human AD using comparative proteomic profiling (Broccardo *et al.* 2011). Future studies will help to elucidate the role of FLG2 in atopic dermatitis. Our results suggest a putative role of FLG2 in cAD.

Skin-specific aspartic peptidase retroviral-like 1 (ASPRV1): ASPRV1 seems to be important for posttranslational processing of profilagrin to filaggrin, a key event during epidermal differentiation (Matsui *et al.* 2011). It was shown that ASPRV1 deficient hairless mice developed dry skin and a thicker and less hydrated stratum corneum. Missense mutations in hAD patients and normal individuals were shown to have a negative effect on the ability of ASPRV1 to cleave the profilaggrin linker peptide (Matsui *et al.* 2011). Another study revealed impaired skin regeneration and remodeling in mice with impaired ASPRV1 expression (Hildenbrand *et al.* 2010). In contrast, a study evaluating atopic Europeans failed to find an association between ASPRV1 gene mutations and atopic eczema (Sandilands *et al.* 2012). The mRNA concentration of ASPRV1 in sensitized dogs after allergen treatment was strongly decreased, in contrast to control dogs. Possibly there is an increased filaggrin demand in allergen-exposed skin that cannot be met by atopic dogs due to a decreased ASPRV1

expression in cAD. This could explain the previous findings in atopic dogs where a loss of function mutation or modified expression of FLG was not present (Chervet *et al.* 2010), but immunohistochemical changes pointing to a filaggrin deficiency were found (Marsella *et al.* 2009).

TGM1 and CE precursor proteins: The protein envelope component of the cornified envelope (CE) consists of different cross-linked proteins (Credille *et al.* 2009). It is assumed that the enzyme transglutaminase 1 (TGM1) is responsible for the crosslinking of CE proteins such as involucrine, loricrin, and others (Greenberg *et al.* 1991; Steinert and Marekov 1995) into a mechanically resistant protein polymer and of attaching lipids (ceramides) to the crosslinked proteins by esterification (Marekov and Steinert 1998; Nemes *et al.* 1999). Neonatal death is seen in mice that lack the *TGM1* gene, which leads to a defective stratum corneum, massive TEWL and subsequent dehydration (Matsuki *et al.* 1998). After skin transplantation from *TGM1* deficient mice to normal ones ichthyosiform changes were observed (Kuramoto *et al.* 2002). Mutations were found in the *TGM1* gene of humans and dogs suffering from ichthyosis (Cao *et al.* 2009; Credille *et al.* 2009). In our study, sensitized dogs showed a diminished expression of TGM1 in allergen PT skin. A disturbed *TGM1* expression may be involved in the development of skin barrier defects characteristic for cAD.

In addition, the sensitized dogs showed a decreased mRNA concentration for a number of different CE precursor proteins including periplakin (PPL), loricrin (LOR) and sciellin (SCEL) in allergen treated skin. The absence of one of the precursor proteins in mice usually does not necessarily lead to clinical consequences (Koch *et al.* 2000; Aho *et al.* 2004; Baden *et al.* 2005): In contrast, mice deficient in three CE Proteins showed an impaired skin barrier und changes in the composition of T cell subpopulations in the skin (Sevilla *et al.* 2007). Kim et al. showed that Th2 cytokines induce the expression of different transcription factors that impede the expression of LOR and involucrin (Kim *et al.* 2008). A skin barrier defect may be present in inflamed skin of these sensitized dogs.

Desmosomal and tight junction proteins: For the mechanical stability of the skin, intercellular connections are important. In the epidermis desmosomes and adherence junctions connect the actin cytoskeleton of adjacent corneocytes. Keratinocyte maturation desmosomes develop into corneodesmosomes that are connected to the CE (Ishida-Yamamoto *et al.* 2011). In the uppermost layer corneodesmosomes are proteolytically degraded allowing the natural scaling of the skin (Serre *et al.* 1991). Desmoplakin (DSP), the intracellular main component of desmosomes belongs to the plakin family, the so-called cyto-linker or desmosomal-linker-proteins. Auto-antibodies against the plakin-family members are known to cause autoimmune mediated skin diseases such as erythema multiforme (Foedinger *et al.* 1995) and epidermolysis bullosa (Jonkmann *et al.* 2005). Together with desmoglein 1 (DSG1) and other proteins, desmoplakin participates in the desmosome formation. *DSP* expression was reduced in allergen PT skin of sensitized dogs. The role of DSP in AD is not elucidated, however a diminished expression may weaken cell to cell-adhesion and subsequently affect barrier function.

The transmembrane protein DSG1, a Ca2+ binding cadherin binds to the DSG1 molecule of the adjacent keratinocyte in desmosomes (Green and Simpson 2007) and corneodesmosomes (Caubet *et al.* 2004; Descargues *et al.* 2006). DSG1 has an important role in the skin barrier and function of the stratum corneum and is decreased in human AD (Broccardo *et al.* 2011), which is in agreement with our findings, that sensitized dogs (in contrast to healthy dogs) showed a decreased gene expression of the 24 h allergen PT compared to saline PT.

The tight junction (TJ) contributes to intercellular adhesion in epithelial cells and is located at the most apical part of their lateral membranes (Farquhar and Palade 1963). In stratified epithelia TJ are located in the maculae occludentes of the stratum granulosum (Squier 1973). The function of the maculae occludentes in the epidermis remains controversial (Hashimoto 1971; Elias and Friend 1975). In mice, TJ's may be involved in the epidermal barrier integrity (Yamamoto *et al.* 2008). Occludin (OCLN) is a transmembrane protein of the TJ (Ando-Akatsuka *et al.* 1996) expressed in the outer layers of the epidermis. Another TJ protein, cingulin (CNG) interacts with other TJ-proteins, such as actin (Bazzoni *et al.* 2000; D'Atri and Citi 2001) and is involved in the regulation of the gene expression of other proteins as well as cell proliferation (Aijaz *et al.* 2005; Guillemot and Citi 2006). In the skin of sensitized dogs CNG and OCLN expression was strongly decreased 24 h after allergen PT. This may contribute to an impaired cell proliferation and TJ formation. Whether defective TJ's are involved the pathophysiology of AD should be examined in further studies.

Protease inhibitors: The balance between cell proliferation, maturation and desquamation is of particular importance for a physiologically sound skin barrier. Endogenous epidermal proteases and exogenous proteases are involved in the process of corneocyte desquamation by corneodesmolysis (Horikoshi *et al.* 1999). Proteases further have the property to activate or inactivate antimicrobial peptides such as cathelicidines in the skin (Yamasaki *et al.* 2006). Protease inhibitors are produced by keratinocytes to prevent excessive protease activity and associated uncontrolled desquamation of the stratum corneum resulting in skin barrier defects and inflammation (Hansson *et al.* 2002; Denecker *et al.* 2008). The gene “serine peptidase inhibitor, Kazal type 5” (*SPINK5*) is coding for an important protease inhibitor. Polymorphisms in this gene have been shown to be associated with human AD (Walley *et al.* 2001; Nishio *et al.* 2003; Weidinger *et al.* 2008) and a loss of function mutation with Netherton syndrome (Chavanas *et al.* 2000). The sensitized dogs in our study showed reduced expression of SPINK5 24 h after allergen PT. On the assumption that SPINK5 in our dog is not mutated a reduced expression would not prevent the skin against excessive protease activitiy and its consequences mentioned before. Another study however, found an increased expression of *SPINK5* in dogs with AD (Wood *et al.* 2009). These contrasting findings could be due to the different biological models used. Wood et al. analyzed skin samples of dogs of different breeds, different age and different disease states. Genes associated with hAD vary between different populations. In dogs it was shown that breed diversity limits the detection of gene association in cAD (Wood *et al.* 2010). In addition it should be mentioned that SNPs or point mutations can lead to dysfunctional proteins without a reduction in expression or sometimes even with increased

expression due to aberrant feedback loops. A reduced SPINK5 expression due to inflammation like in our study or a lack of SPINK5 function due to a loss of function mutation with normal or increased expression may be involved in cAD in some breeds and protease inhibitors could be a new therapeutic option (Egelrud *et al.* 2005).

Further DEGs

Keratinocyte proline-rich protein (*KPRP*) is a recently identified marker of epidermal differentiation (Kong *et al.* 2003). KPRP in humans is expressed in the stratum granulosum and Lee et al. 2005 found an increased expression in patients with psoriasis. In contrast its expression in patients with hAD was decreased (Lee *et al.* 2005). A decreased expression of KPRP in sensitized compared to control dogs was found in this study and indicates a participation of *KPRP* in the pathophysiology of cAD. It is not known if KPRP is a structural protein of the cornified envelope or has other function with regard to skin differentiation, thus further studies are needed to elucidate its role in the cutaneous homeostasis.

Calmodulin-like 5 (*CALML5*) is an epidermal protein related to the calmodulin family of Ca^{2+}-binding proteins. CALML5 is highly expressed during keratinocyte differentiation and an increased concentration of *CALML5* was found in the skin of psoriasis patients, although it was not clear if this increase was due to an enhanced expression or reduced proteolytic degradation (Mehul *et al.* 2001). The diminished expression of *CALML5* in the skin of sensitized dogs 24 h after allergen PT may indicate disturbed epidermal differentiation.

Peroxisome proliferator-activated receptor alpha (*PPARA*) is a transcription factor activated by fatty acids that are produced in inflammation (Moraes *et al.* 2006). In the skin, *PPARA* is expressed by keratinocytes (Rivier *et al.* 1998), Langerhans cells (Dubrac *et al.* 2007), macrophages (Babaev *et al.* 2007) and T-cells (Cunard *et al.* 2002). *PPARA* regulates the proliferation and differentiation of keratinocytes (Komuves *et al.* 2000) and is involved in wound healing (Michalik *et al.* 2001). In human atopic skin a diminished expression of *PPARA* was documented (Plager *et al.* 2007). The activation of *PPARA* by topical treatment with PPARA-ligands showed anti-inflammatory effects in humans with AD (Eberlein *et al.* 2008; Eichenfield *et al.* 2009) and ultraviolet-B-light-induced skin inflammation (Kippenberger *et al.* 2001). Törma et al. showed that *PPARA* expression decreases early in skin inflammation following allergen exposure (Törma and Berne 2009). In the present study, sensitized dogs showed a decrease of PPARA expression 24 h after allergen PT in comparison to the control group. As *PPARA* is not only involved in keratinocyte differentiation, but also has regulatory activity in skin inflammation (Dubrac and Schmuth 2011). The importance of PPARA-ligands in cAD should be elucidated. Arachidone lipoxygenase 3 (*ALOXE3*), which codes for the LOX3 protein, which is predominantly expressed in the epidermis (Krieg *et al.* 2002). The enzymes of *ALOXE3* and *ALOX12B* convert arachidonic acid into epoxyalcohol products, which activate *PPARA* and therefore seem to play an important role in epidermal differentiation (Yu *et al.* 2007). Functional impairment of either *ALOX12B* or *ALOXE3* results in ichthyosiform skin disease in humans (Jobard *et al.* 2002). Expression of *ALOXE3* also decreases skin inflammation after

allergen exposure (Törmä and Berne 2009). Our results show, for the first time, a similar expression pattern of *PPARA* and *ALOXE3* in canine skin inflammation. We hypothesize that both genes play a role in cAD.

References

Abboud, G., D. Staumont-Salle, A. Kanda, T. Roumier, N. Deruytter *et al.,* 2009 Fc(epsilon)RI and FcgammaRIII/CD16 differentially regulate atopic dermatitis in mice. J. Immunol. 182: 6517-6526.

Aho, S., K. Li, Y. Ryoo, C. McGee, A. Ishida-Yamamoto *et al.*, 2004 Periplakin gene targeting reveals a constituent of the cornified cell envelope dispensable for normal mouse development. Mol. Cell Biol. 24: 6410-6418.

Aijaz, S., F. D'Atri, S. Citi, M. S. Balda, and K. Matter, 2005 Binding of GEF-H1 to the tight junction-associated adaptor cingulin results in inhibition of Rho signaling and G1/S phase transition. Dev. Cell 8: 777-786.

Ando-Akatsuka, Y., M. Saitou, T. Hirase, M. Kishi, and A. Sakakibara *et al.*, 1996 Interspecies diversity of the occludin sequence: cDNA cloning of human, mouse, dog, and rat-kangaroo homologues. J. Cell Biol. 133: 43-47.

Arakawa, S., Y. Hatano, and K. Katagiri, 2004 Differential expression of mRNA for Th1 and Th2 cytokine-associated transcription factors and suppressors of cytokine signalling in peripheral blood mononuclear cells of patients with atopic dermatitis. Clin. Exp. Immunol. 135: 505-510.

Arshad, S. H., W. Karmaus, R. Kurukulaaratchy, A. Sadeghnejad, M. Huebner *et al.*, 2008 Polymorphisms in the interleukin 13 and GATA binding protein 3 genes and the development of eczema during childhood. Br. J. Dermatol. 158: 1315-1322.

Babaev, V. R., H. Ishiguro, L. Ding, P. G. Yancey, D. E. Dove *et al.*, 2007 Macrophage expression of peroxisome proliferator-activated receptor-alpha reduces atherosclerosis in low-density lipoprotein receptor-deficient mice. Circulation 116: 1404-1412.

Baden, H. P., M. F. Champliaud, J. P. Sundberg, and A. Viel, 2005 Targeted deletion of the sciellin gene resulted in normal development and maturation. Genesis 42: 219-228.

Bae, C. J., J. W. Lee, S. B. Shim, S. W. Jee, S. H. Lee *et al.*, 2011 GATA binding protein 3 overexpression and suppression significantly contribute to the regulation of allergic skin inflammation. Int. J. Mol. Med. 28: 171-179.

Banchereau, J., and R. M. Steinman, 1998 Dendritic cells and the control of immunity. Nature 392: 245-252.

Barker, J. N., C. N. Palmer, Y. Zhao, H. Liao, P. R. Hull *et al.*,2007 Null mutations in the filaggrin gene (FLG) determine major susceptibility to early-onset atopic dermatitis that persists into adulthood. J. Invest. Dermatol. 127: 564-567.

Barros Roque, J., C. A. O'Leary, M. Kyaw-Tanner, M. Latter, K. Mason *et al.*, 2009 Haplotype sharing excludes canine orthologous Filaggrin locus in atopy in West Highland White Terriers. Anim. Genet. 40: 793-4.

Baurecht, H., A. D. Irvine, N. Novak, T. Illig, B. Buhler *et al.*, 2007. Toward a major risk factor for atopic eczema: meta-analysis of filaggrin polymorphism data. J. Allergy Clin. Immunol. 120: 1406-1412.

Bazzoni, G., O. M. Martinez-Estrada, F. Orsenigo, M. Cordenonsi, S. Citi *et al.*, 2000 Interaction of junctional adhesion molecule with the tight junction components ZO-1, cingulin, and occludin. J. Biol. Chem. 275: 20520-20526.

Berne, R. M., R. M. Knabb, S. W. Ely, and R. Rubio, 1983 Adenosine in the local regulation of blood flow: a brief overview. Fed. Proc. 42: 3136-3142.

Brasier, A. R., M. Lu, T. Hai, Y. Lu, and I. Boldogh, 2001 NF-kappa B-inducible BCL-3 expression is an autoregulatory loop controlling nuclear p50/NF-kappa B1 residence. J. Biol. Chem. 276: 32080-32093.

Broccardo, C.J., S. Mahaffey, J. Schwarz, L. Wruck, G. David et al., 2011 Comparative proteomic profiling of patients with atopic dermatitis based on history of eczema herpeticum infection and Staphylococcus aureus colonization. J. Allergy Clin. Immunol. 127: 186-193.

Buchau, A. S., D. T. MacLeod, S. Morizane, P. F. Kotol, T. Hata *et al.*, 2009 Bcl-3 acts as an innate immune modulator by controlling antimicrobial responses in keratinocytes. J. Invest. Dermatol. 129: 2148-2155.

Cao, X., Z. Lin, H. Yang, D. Bu, P. Tu *et al.*, 2009 New mutations in the transglutaminase 1 gene in three families with lamellar ichthyosis. Clin. Exp. Dermatol. 34: 904-909.

Caubet, C., N. Jonca, M. Brattsand, M. Guerrin, D. Bernard *et al.*, 2004 Degradation of corneodesmosome proteins by two serine proteases of the kallikrein family, SCTE/KLK5/hK5 and SCCE/KLK7/hK7. J. Invest. Dermatol. 122: 1235-1244.

Chang, N. C., S. I. Hung, K. Y. Hwa, I. Kato, J. E. Chen *et al.*, 2001 A macrophage protein, Ym1, transiently expressed during inflammation is a novel mammalian lectin. J. Biol. Chem. 276: 17497-17506.

Chavanas, S., C. Garner, C. Bodemer, M. Ali, D. H. Teillac, J. Wilkinson *et al.*, 2000 Localization of the Netherton syndrome gene to chromosome 5q32, by linkage analysis and homozygosity mapping. Am. J. Hum. Genet. 66: 914-921.

Chen, Y., S. Lind Enoksson, C. Johansson, M. A. Karlsson, L. Lundeberg *et al.*, 2011 The expression of BAFF, APRIL and TWEAK is altered in eczema skin but not in the circulation of atopic and seborrheic eczema patients. PLoS One 6: e22202.

Chervet, L., A. Galichet, W. H. McLean, H. Chen, M. M. Suter *et al.*, 2010 Missing C-terminal filaggrin expression, NFkappaB activation and hyperproliferation identify the dog as a putative model to study epidermal dysfunction in atopic dermatitis. Exp. Dermatol. 19: e343-346.

Chomarat, P., and J. Banchereau, 1998 Interleukin-4 and interleukin-13: their similarities and discrepancies. Int. Rev. Immunol. 17: 1-52.

Chupp, G. L., C. G. Lee, N. Jarjour, Y. M. Shim, C. T. Holm *et al.*, 2007 A chitinase-like protein in the lung and circulation of patients with severe asthma. N. Engl. J. Med. 357: 2016-2027.

Credille, K. M., J. S. Minor, K. F. Barnhart, E. Lee, M. L. Cox et al. 2009 Transglutaminase 1-deficient recessive lamellar ichthyosis associated with a LINE-1 insertion in Jack Russell terrier dogs. Br. J. Dermatol. 161: 265-272.

Cunard, R., D. DiCampli, D. C. Archer, J. L. Stevenson, M. Ricote *et al.*, 2002 WY14,643, a PPAR alpha ligand, has profound effects on immune responses in vivo. J. Immunol. 169: 6806-6812.

D'Atri F., and S. Citi, 2001 Cingulin interacts with F-actin in vitro. FEBS Lett. 507: 21-24.

Da Silva, C. A., D. Hartl, W. Liu, C. G. Lee, and J. A. Elias, 2008 TLR-2 and IL-17A in chitin-induced macrophage activation and acute inflammation. J. Immunol. 181: 4279-4286.

Debes, G. F., and M. C. Diehl, 2011 CCL8 and skin T cells - an allergic attraction. Nat. Immunol. 12: 111-112.

DeBoer, D.J., 1998 In vitro production of IgG4 by peripheral blood mononuclear cells (PBMC): the contribution of committed B cells. Clin. Exp. Immunol. 114: 252-257.

Denecker, G., P. Ovaere, P. Vandenabeele, and W. Declercq, 2008 Caspase-14 reveals its secrets. J. Cell Biol. 180: 451-458.

Descargues, P., C. Deraison, C. Prost, S. Fraitag, J. Mazereeuw-Hautier *et al.*, 2006 Corneodesmosomal cadherins are preferential targets of stratum corneum trypsin- and chymotrypsin-like hyperactivity in Netherton syndrome. J. Invest. Dermatol. 126: 1622-1632.

Driver, A. G., C. A. Kukoly, S. Ali, and S. J. Mustafa, 1993 Adenosine in bronchoalveolar lavage fluid in asthma. Am. Rev. Respir. Dis. 148: 91-97.

Dubrac, S., P. Stoitzner, D. Pirkebner, A. Elentner, K. Schoonjans *et al.*, 2007 Peroxisome proliferator-activated receptor-alpha activation inhibits Langerhans cell function. J. Immunol. 178: 4362-4372.

Dubrac, S., and M. Schmuth, 2011 PPAR-alpha in cutaneous inflammation. Dermatoendocrinol. 3: 23-26.

Eberlein, B., C. Eicke, H. W. Reinhardt, and J. Ring, 2008 Adjuvant treatment of atopic eczema: assessment of an emollient containing N-palmitoylethanolamine (ATOPA study). J. Eur. Acad. Dermatol. Venereol. 22: 73-82.

Egelrud, T., M. Brattsand, P. Kreutzmann, M. Walden, K. Vitzithum *et al.*, 2005 hK5 and hK7, two serine proteinases abundant in human skin, are inhibited by LEKTI domain 6. Br. J. Dermatol. 153: 1200-1203.

Eichenfield, L. F., A. McCollum, and P. Msika, 2009 The benefits of sunflower oleodistillate (SOD) in pediatric dermatology. Pediatr. Dermatol. 26: 669-675.

Elias, P.M., and D. S. Friend, 1975 The permeability barrier in mammalian epidermis. J. Cell Biol. 65: 180-191.

Elliott, J., and J. A. Johnston, 2004 SOCS: role in inflammation, allergy and homeostasis. Trends Immunol. 25: 434-440.

Farquhar, M. G., and G. E. Palade, 1963 Junctional complexes in various epithelia. J. Cell Biol. 17: 375-412.

Foedinger, D., G. J. Anhalt, B. Boecskoer, A. Elbe, K. Wolff *et al.*, 1995 Autoantibodies to desmoplakin I and II in patients with erythema multiforme. J. Exp. Med. 181: 169-179.

Giustizieri, M. L., F. Mascia, A. Frezzolini, O. De Pita, L. M. Chinni et al., 2001 Keratinocytes from patients with atopic dermatitis and psoriasis show a distinct chemokine production profile in response to T cell-derived cytokines. J. Allergy Clin. Immunol. 107: 871-877.

Green, K. J., and C. L. Simpson, 2007 Desmosomes: new perspectives on a classic. J. Invest. Dermatol. 127: 2499-2515.

Greenberg, C. S., P. J. Birckbichler, and R. H. Rice, 1991 Transglutaminases: multifunctional cross-linking enzymes that stabilize tissues. FASEB J. 5: 3071-3077.

Guillemot, L., and S. Citi, 2006 Cingulin regulates claudin-2 expression and cell proliferation through the small GTPase RhoA. Mol. Biol. Cell 17: 3569-3577.

Halliwell, R. E. W., and D. J. DeBoer. 2001 The ACVD task force on canine atopic dermatitis (III): the role of antibodies in canine atopic dermatitis. Vet. Immunol. Immunopathol. 81: 159-167.

Hamid, Q., T. Naseer, E. M. Minshall, Y. L. Song, M. Boguniewicz *et al.*, 1996 In vivo expression of IL-12 and IL-13 in atopic dermatitis. J. Allergy Clin. Immunol. 98: 225-231.

Hansson, L., A. Backman, A. Ny, M. Edlund, E. Ekholm et al., 2002 Epidermal overexpression of stratum corneum chymotryptic enzyme in mice: a model for chronic itchy dermatitis. J. Invest. Dermatol. 118: 444-449.

Hashimoto, K., 1971 Intercellular spaces of the human epidermis as demonstrated with lanthanum. J. Invest. Dermatol. 57: 17-31.

Hildenbrand, M., V. Rhiemeier, B. Hartenstein, B. Lahrmann, N. Grabe *et al.*, 2010 Impaired skin regeneration and remodeling after cutaneous injury and chemically induced hyperplasia in taps-transgenic mice. J. Invest. Dermatol. 130: 1922-1930.

Homey, B., M. Steinhoff, T. Ruzicka, and D. Y. Leung, 2006 Cytokines and chemokines orchestrate atopic skin inflammation. J. Allergy Clin. Immunol. 118: 178-189.

Horikoshi, T., S. Igarashi, H. Uchiwa, H. Brysk, and M. M. Brysk, 1999 Role of endogenous cathepsin D-like and chymotrypsin-like proteolysis in human epidermal desquamation. Br. J. Dermatol. 141: 453-459.

Hussein, Y. M., A. S. Ahmad, M. M. Ibrahem, H. M. Elsherbeny, S. M. Shalaby *et al.*, 2011 Interleukin 13 receptors as biochemical markers in atopic patients. J. Investig. Allergol. Clin. Immunol. 21: 101-107.

Ishida-Yamamoto, A., S. Igawa, and M. Kishibe, 2011 Order and disorder in corneocyte adhesion. J. Dermatol. 38: 645-654.

Jee, H. M., K. W. Kim, J. Y. Hong, M. H. Sohn, and K. E. Kim, 2010 Increased serum B cell-activating factor level in children with atopic dermatitis. Clin. Exp. Dermatol. 35: 593-598.

Jobard, F., C. Lefevre, A. Karaduman, C. Blanchet-Bardon, S. Emre *et al.*, 2002 Lipoxygenase-3 (ALOXE3) and 12(R)-lipoxygenase (ALOX12B) are mutated in non-bullous congenital ichthyosiform erythroderma (NCIE) linked to chromosome 17p13.1. Hum. Mol. Genet. 11: 107-113.

Jonkman, M. F., A. M. Pasmooij, S. G. Pasmans, M. P. van den Berg, H. J. Ter Horst *et al.*, 2005 Loss of desmoplakin tail causes lethal acantholytic epidermolysis bullosa. Am. J. Hum. Genet. 77: 653-660.

Kaburagi, Y., Y. Shimada, T. Nagaoka, M. Hasegawa, K. Takehara *et al.*, 2001 Enhanced production of CC-chemokines (RANTES, MCP-1, MIP-1alpha, MIP-1beta, and eotaxin) in patients with atopic dermatitis. Arch. Dermatol. Res. 293: 350-355.

Kang, J. S., Y. D. Yoon, J. H. Ahn, S. C. Kim, K. H. Kim *et al.*, 2006 B cell-activating factor is a novel diagnosis parameter for asthma. Int. Arch. Allergy Immunol. 141: 181-188.

Katagiri, K., S. Itami, Y. Hatano, and S. Takayasu, 1997 Increased levels of IL-13 mRNA, but not IL-4 mRNA, are found in vivo in peripheral blood mononuclear cells (PBMC) of patients with atopic dermatitis (AD). Clin. Exp. Immunol. 108: 289-294.

Kiekens, R. C., T. Thepen, I. C. Bihari, E. F. Knol, J. G. Van De Winkel *et al.*, 2000 Expression of Fc receptors for IgG during acute and chronic cutaneous inflammation in atopic dermatitis. Br. J. Dermatol. 142: 1106-1113.

Kim, B. E., D. Y. Leung, M. Boguniewicz, and M. D. Howell. Loricrin and involucrin expression is down-regulated by Th2 cytokines through STAT-6. Clin. Immunol. 126: 332-337.

Kinet, J.P., 1999 The high-affinity IgE receptor (Fc epsilon RI): from physiology to pathology. Ann. Rev. Immunol. 17: 931-972.

Kippenberger, S., S. M. Loitsch, M. Grundmann-Kollmann, S. Simon, T. A. Dang *et al.*, 2001 Activators of peroxisome proliferator-activated receptors protect human skin from ultraviolet-B-light-induced inflammation. J. Invest. Dermatol. 117: 1430-1436.

Koch, P. J., P. A. de Viragh, E. Scharer, D. Bundman, M. A. Longley *et al.*, 2000 Lessons from loricrin-deficient mice: compensatory mechanisms maintaining skin barrier function in the absence of a major cornified envelope protein. J. Cell Biol. 151: 389-400.

Komuves, L. G., K. Hanley, A. M. Lefebvre, M. Q. Man, D. C. Ng *et al.*, 2000 Stimulation of PPARalpha promotes epidermal keratinocyte differentiation in vivo. J. Invest. Dermatol. 115: 353-360.

Kong, W., M. T. Longaker, and H. P. Lorenz, 2003 Molecular cloning and expression of keratinocyte proline-rich protein, a novel squamous epithelial marker isolated during skin development. J. Biol. Chem. 278: 22781-222786.

Krieg, P., M. Heidt, M. Siebert, A. Kinzig, F. Marks *et al.*, 2002 Epidermis-type lipoxygenases. Adv. Exp. Med. Biol. 507: 165-170.

Kuramoto, N., T. Takizawa, T. Takizawa, M. Matsuki, H. Morioka *et al.*, 2002 Development of ichthyosiform skin compensates for defective permeability barrier function in mice lacking transglutaminase 1. J. Clin. Invest. 109: 243-250.

Langstein, J., J. Michel, J. Fritsche, M. Kreutz, R. Andreesen *et al.*, 1998 CD137 (ILA/4-1BB), a member of the TNF receptor family, induces monocyte activation via bidirectional signaling. J. Immunol. 160: 2488-2494.

Lee, C. G., C. A. Da Silva, J. Y. Lee, D. Hartl, and J. A. Elias, 2008 Chitin regulation of immune responses: an old molecule with new roles. Curr. Opin. Immunol. 20: 684-689.

Lee, C. G., 2009 Chitin, chitinases and chitinase-like proteins in allergic inflammation and tissue remodeling. Yonsei Med. J. 50: 22-30.

Lee, C. G., D. Hartl, G. R. Lee, B. Koller, H. Matsuura *et al.*, 2009 Role of breast regression protein 39 (BRP-39)/chitinase 3-like-1 in Th2 and IL-13-induced tissue responses and apoptosis. J. Exp. Med. 206: 1149-1166.

Lee, W. H., S. Jang, J. S. Lee, Y. Lee, E. Y. Seo *et al.*, 2005 Molecular cloning and expression of human keratinocyte proline-rich protein (hKPRP), an epidermal marker isolated from calcium-induced differentiating keratinocytes. J. Invest. Dermatol. 125: 995-1000.

Leung, D. Y., M. Boguniewicz, M. D. Howell, I. Nomura, and Q. A. Hamid, 2004 New insights into atopic dermatitis. J. Clin. Invest. 113: 651-657.

Lian, T.M., and R. E. W. Halliwell, 1998 Allergen-specific IgE and IgGd antibodies in atopic and normal dogs. Vet. Immunol. Immunopathol. 66: 203-223.

Liew, F. Y., N. I. Pitman, and I. B. McInnes, 2010 Disease-associated functions of IL-33: the new kid in the IL-1 family. Nat. Rev. Immunol. 10: 103-10.

Mackay, F., and P. Schneider, 2009 Cracking the BAFF code. Nat. Rev. Immunol. 9: 491-502.

Marekov, L. N., and P. M. Steinert, 1998 Ceramides are bound to structural proteins of the human foreskin epidermal cornified cell envelope. J. Biol. Chem. 273: 17763-17770.

Marenholz, I., V. A. Rivera, J. Esparza-Gordillo, A. Bauerfeind, M. A. Lee-Kirsch et al., 2011 Association screening in the Epidermal Differentiation Complex (EDC) identifies an SPRR3 repeat number variant as a risk factor for eczema. J. Invest. Dermatol. 131: 1644-1649.

Marquardt, D. L., and L. L. Walker, 1990 Modulation of mast cell responses to adenosine by agents that alter protein kinase C activity. Biochem. Pharmacol. 39: 1929-1934.

Marsella, R., D. Samuelson, and L. Harrington, 2009 Immunohistochemical evaluation of filaggrin polyclonal antibody in atopic and normal beagles. Vet. Dermatol. 20: 547-554.

Massoumi, R., K. Chmielarska, K. Hennecke, A. Pfeifer and R. Fassler, 2006 Cyld inhibits tumor cell proliferation by blocking Bcl-3-dependent NF-kappaB signaling. Cell 125: 665-677.

Matsui, T., K. Miyamoto, A. Kubo, H. Kawasaki, T. Ebihara T, et al., 2011 SASPase regulates stratum corneum hydration through profilaggrin-to-filaggrin processing. EMBO Mol. Med. 3: 320-333.

Matsuki, M., F. Yamashita, A. Ishida-Yamamoto, K. Yamada, C. Kinoshita *et al.*, 1998 Defective stratum corneum and early neonatal death in mice lacking the gene for transglutaminase 1 (keratinocyte transglutaminase). Proc. Nat. Acad. Sci. U. S. A. 95: 1044-1049.

McKeithan, T.W., H. Ohno, and M. O. Diaz, 1990 Identification of a transcriptional unit adjacent to the breakpoint in the 14;19 translocation of chronic lymphocytic leukemia. Genes Chromosomes Cancer 1: 247-255.

Mehul, B., D. Bernard,and R. Schmidt, 2001 Calmodulin-like skin protein: a new marker of keratinocyte differentiation. J. Invest. Dermatol. 116: 905-909.

Michalik, L., B. Desvergne, N. S. Tan, S. Basu-Modak, P. Escher *et al.*, 2001 Impaired skin wound healing in peroxisome proliferator-activated receptor (PPAR)alpha and PPARbeta mutant mice. J. Cell Biol. 154: 799-814.

Moraes, L.A., L. Piqueras, and D. Bishop-Bailey, 2006 Peroxisome proliferator-activated receptors and inflammation. Pharmacol. Ther. 110: 371-385.

Nawijn, M.C., G. M. Dingjan, R. Ferreira, B. N. Lambrecht, A. Karis *et al.*, 2001 Enforced expression of GATA-3 in transgenic mice inhibits Th1 differentiation and induces the formation of a T1/ST2-expressing Th2-committed T cell compartment in vivo. J. Immunol. 167: 724-732.

Nemes, Z., L. N. Marekov, L. Fesus L, and P. M. Steinert, 1999 A novel function for transglutaminase 1: attachment of long-chain omega-hydroxyceramides to involucrin by ester bond formation. Proc. Nat. Acad. Sci. U. S. A. 96: 8402-8407.

Nemoto-Hasebe, I., M. Akiyama, T. Nomura, A. Sandilands, W. H. McLean *et al.*, 2009 FLG mutation p.Lys4021X in the C-terminal imperfect filaggrin repeat in Japanese patients with atopic eczema. Br. J. Dermatol. 161: 1387-1390.

Nishio, Y., E. Noguchi, M. Shibasaki, M. Kamioka, E. Ichikawa et al., 2003 Association between polymorphisms in the SPINK5 gene and atopic dermatitis in the Japanese. Genes Immun. 4: 515-517.

Ober, C., and G. L. Chupp, 2009 The chitinase and chitinase-like proteins: a review of genetic and functional studies in asthma and immune-mediated diseases. Curr. Opin. Allergy Clin. Immunol. 9: 401-408.

Plager, D. A., A. A. Leontovich, S. A. Henke, M. D. Davis, M. T. McEvoy *et al.*, 2007 Early cutaneous gene transcription changes in adult atopic dermatitis and potential clinical implications. Exp. Dermatol. 16: 28-36.

Prefontaine, D., S. Lajoie-Kadoch, S. Foley, S. Audusseau, R. Olivenstein et al., 2009 Increased expression of IL-33 in severe asthma: evidence of expression by airway smooth muscle cells. J. Immunol. 183: 5094-5103.

Ravetch, J. V., and J. P. Kinet JP, 1991 Fc receptors. Ann. Rev. Immunol. 9: 457-492.

Reese, T. A., H. E. Liang, A. M. Tager, A. D. Luster, N. Van Rooijen *et al.*, 2007 Chitin induces accumulation in tissue of innate immune cells associated with allergy. Nature 447: 92-96.

Renkema, G.H., R. G. Boot, F. L. Au, W. E. Donker-Koopman, A. Strijland *et al.*, 1998 Chitotriosidase, a chitinase, and the 39-kDa human cartilage glycoprotein, a chitin-binding lectin, are homologues of family 18 glycosyl hydrolases secreted by human macrophages. Eur. J. Biochem. 251: 504-509.

Rivier, M., I. Safonova, P. Lebrun, C. E. Griffiths, G. Ailhaud *et al.*, 1998 Differential expression of peroxisome proliferator-activated receptor subtypes during the differentiation of human keratinocytes. J. Invest. Dermatol. 111: 1116-1121.

Rorke, S., and S. T. Holgate. Targeting adenosine receptors: novel therapeutic targets in asthma and chronic obstructive pulmonary disease. Am. J. Respir. Med. 1: 99-105.

Sandilands, A., S. J. Brown, C. S. Goh, E. Pohler, N. J. Wilson *et al.*, 2012 Mutations in the SASPase gene (ASPRV1) are not associated with atopic eczema or clinically dry skin. J. Invest. Dermatol. 132: 1507-1510.

Schlotter, Y. M., V. P. Rutten, F. M. Riemers, E. M. Knol, and T. Willemse, 2011 Lesional skin in atopic dogs shows a mixed Type-1 and Type-2 immune responsiveness. Vet. Immunol. Immunopathol. 143: 20-26.

Schwarz, H., J. Valbracht, J. Tuckwell, J. von Kempis, and M. Lotz, 1995 ILA, the human 4-1BB homologue, is inducible in lymphoid and other cell lineages. Blood 85: 1043-1052.

Seki, Y., H. Inoue, N. Nagata, K. Hayashi, S. Fukuyama *et al.*, 2003 SOCS-3 regulates onset and maintenance of T(H)2-mediated allergic responses. Nat. Med. 9: 1047-1054.

Serre, G., V. Mils, M. Haftek, C. Vincent, F. Croute *et al.*, 1991 Identification of late differentiation antigens of human cornified epithelia, expressed in re-organized desmosomes and bound to cross-linked envelope. J. Invest. Dermatol. 97: 1061-1072.

Sevilla, L.M., R. Nachat, K. R. Groot, J. F. Klement, J. Uitto *et al.*, 2007 Mice deficient in involucrin, envoplakin, and periplakin have a defective epidermal barrier. J. Cell Biol. 179: 1599-1612.

Shibata, Y., W. J. Metzger, and Q. N. Myrvik, 1997 Chitin particle-induced cell-mediated immunity is inhibited by soluble mannan: mannose receptor-mediated phagocytosis initiates IL-12 production. J. Immunol. 159: 2462-2467.

Sicherer, S.H., and D. Y. Leung, 2006 Advances in allergic skin disease, anaphylaxis, and hypersensitivity reactions to foods, drugs, and insects. J. Allergy Clin. Immunol. 118: 170-177.

Sohn, M. H., J. H. Lee, K. W. Kim, S. W. Kim, S. H. Lee *et al.*, 2009 Genetic variation in the promoter region of chitinase 3-like 1 is associated with atopy. Am. J. Respir. Crit. Care Med. 179: 449-456.

Squier, C. A., 1973 The permeability of keratinized and nonkeratinized oral epithelium to horseradish peroxidase. J. Ultrastruct. Res. 43: 160-177.

Steinert, P. M., and L. N. Marekov, 1995 The proteins elafin, filaggrin, keratin intermediate filaments, loricrin, and small proline-rich proteins 1 and 2 are isodipeptide cross-linked components of the human epidermal cornified cell envelope. J. Biol. Chem. 270: 17702-17711.

Suttner, K., M. Depner, N. Klopp, T. Illig, C. Vogelberg *et al.*, 2009 Genetic variants in the GATA3 gene are not associated with asthma and atopic diseases in German children. J. Allergy Clin. Immunol. 123: 1179-1181.

Taha, R. A., E. M. Minshall, D. Y. Leung, M. Boguniewicz, A. Luster et al., 2000 Evidence for increased expression of eotaxin and monocyte chemotactic protein-4 in atopic dermatitis. J. Allergy Clin. Immunol. 105: 1002-1007.

Tang, L., 2001 Molecular cloning of canine IL-13 receptor alpha chain (alpha1 and alpha2) cDNAs and detection of corresponding mRNAs in canine tissues. Vet. Immunol. Immunopathol. 79: 181-195.

Törmä, H., and B. Berne, 2009 Sodium lauryl sulphate alters the mRNA expression of lipid-metabolizing enzymes and PPAR signalling in normal human skin in vivo. Exp. Dermatol. 18: 1010-1015.

Walley, A.J., S. Chavanas, M. F. Moffatt, R. M. Esnouf, B. Ubhi et al., 2001 Gene polymorphism in Netherton and common atopic disease. Nat. Genet. 29: 175-178.

Weidinger, S., H. Baurecht, S. Wagenpfeil, J. Henderson, N. Novak et al., 2008 Analysis of the individual and aggregate genetic contributions of previously identified serine peptidase inhibitor Kazal type 5 (SPINK5), kallikrein-related peptidase 7 (KLK7), and filaggrin (FLG) polymorphisms to eczema risk. J. Allergy Clin. Immunol. 122: 560-568.

Willemse, A., A. Noordzij, W. E. Van den Brom, and V. P. Rutten, 1985 Allergen specific IgGd antibodies in dogs with atopic dermatitis as determined by the enzyme linked immunosorbent assay (ELISA). Clin. Exp. Immunol. 59: 359-363.

Wood, S. H., D. N. Clements, W. E. Ollier, T. Nuttall, N. A. McEwan et al., 2009 Gene expression in canine atopic dermatitis and correlation with clinical severity scores. J. Dermatol. Sci. 55: 27-33.

Wood, S. H., W. E. Ollier, T. Nuttall, N. A. McEwan, and S. D. Carter, 2010 Despite identifying some shared gene associations with human atopic dermatitis the use of multiple dog breeds from various locations limits detection of gene associations in canine atopic dermatitis. Vet. Immunol. Immunopathol. 138: 193-197.

Wu, C., H. Guo, Y. Wang, Y. Gao, Z. Zhu *et al.*, 2011 Extracellular domain of human 4-1BBL enhanced the function of cytotoxic T-lymphocyte induced by dendritic cell. Cell Immunol. 271: 118-123.

Wu, Z., B. Hansmann, U. Meyer-Hoffert, R. Glaser, and J. M. Schroder, 2009 Molecular identification and expression analysis of filaggrin-2, a member of the S100 fused-type protein family. PLoS One 4: e5227.

Yamamoto, T., M. Kurasawa, T. Hattori, T. Maeda, H. Nakano *et al.*, 2008 Relationship between expression of tight junction-related molecules and perturbed epidermal barrier function in UVB-irradiated hairless mice. Arch. Dermatol. Res. 300: 61-68.

Yamasaki, K., J. Schauber, A. Coda, H. Lin, R. A. Dorschner et al., 2006 Kallikrein-mediated proteolysis regulates the antimicrobial effects of cathelicidins in skin. FASEB J. 20: 2068-2080.

Yu, Z., C. Schneider, W. E. Boeglin, and A. R. Brash. Epidermal lipoxygenase products of the hepoxilin pathway selectively activate the nuclear receptor PPARalpha. Lipids 42: 491-497.

Zheng, W., and R. A. Flavell, 1997 The transcription factor GATA-3 is necessary and sufficient for Th2 cytokine gene expression in CD4 T cells. Cell 89: 587-596.

Table S1 Design of specimen collection; each biopsy was cut into three pieces - one of these was used for histological analysis, the other two parts were used for microarray analysis

day	6 sensitized dogs	6 non sensitized dogs
4 days before patch test	clipping the left lateral thorax	
before patch test (0h)	1 biopsy (non treated skin) / dog	
patch test	patch test with allergen*/saline	patch test with allergen*/saline
+6 h	1 biopsy of each treatment-area (allergen and saline = 2 biopsies / dog)	1 biopsy of each treatment-area (allergen and saline = 2 biopsies / dog)
+24 h	1 biopsy of each treatment area (allergen and saline = 2 biopsies / dog)	1 biopsy of each treatment area (allergen and saline = 2 biopsies / dog)
number of taken biopsies	30	30

Table S2 Sequences of the oligonucleotides used as primers for qPCR

GeneID	Accession Number	Forward Primer	Reverse Primer
[a]RPL13A	XM_533620	CTGCCCCACAAGACCAAG	GGGATCCCATCAAACACCT
[a]LOC479750	XM_536878	GCAGGAAGGGATTCTCCAG	GGTCCAGTAAGAAATCTTCCATAA
[a]UBB	XM_858590	CGCGGCTGGCATTGTTAGCTG	GCATACCACCCCTCAGGCGC
ALOXE3	XM_546605	GCACCTGTGGCCAGAGCCCTA	CAGCTCCGTGGAGCAGCACAC
CCL8	NM_001005255	TGCCAGCTTCAGCACCTTTGTCC	TGGGGTCAGCACAGATCTCCCTG
CLEC7A	XM_849050	TCAAGGCATCACTGGGCGACCT	TCACAGCAATGGGACGCCAACG
DMKN	XM_533694	CTGGGGGTCAGGGCTTCGGA	TCCTCCACTGGACCCGTGCC
DSG1	NM_001002939	CCCAGCAGCGATGAACTGGCA	TCACGACAGGCTGCAGCGAA
DSP	XM_545329	TTCTGCAGGAGGAGGGCACCC	GTGGCCTTCAGGCGAGTGAGC
FCGR3A	XM_536141	TGGCTGCACAATGGGAGACCCAT	GAGGGACCTGGAGCAAGAGCCA
FLG2	XM_540329	TGGCACACTGAGCAAGGATGAGC	GCTGAGAACCTTGTTGCAGGCCA
IL13RA2	NM_001003075	TGGAAACCTGGCATGGGTGTCC	TCTGCTGAATGGTCCAAGCCCTCA
IL18BP	NM_001048018	GCCTCTCCTGGCTCCGGACA	CCGACCTGGGAGGTGCTCGA
IL33	NM_001003180	TTTGCTGCATGCCAACAACGAGG	AGGAAGAAGGCCTGGTCTGGCAA
KPRP	ENSCAFT00000020644	AAGTCCCTGTCCACGTCCTGCT	CGTGGCTCGGGAAATTCACGCT
LOC476953	XM_534152	CAGCACCAGCATCCCAGCTCC	CCCGTGCTTGGGATGGCACT
OCLN	NM_001003195	GCTCTGGGATCCTGCTCGTCCT	CGTGCATGTCCCCACCGTACAC

PPARA	NM_001003093	AAAGCCCGGGTCATCCTCGC	GCGCACCTCTGCCTCCTTGTT
SOCS3	NM_001031631	TGAACGCAGTGTGCAAGCTGC	AGCGTGAAGAAGTGGCGCTGG
SPINK5	NM_001025397	GCGTGGCCCAGATGGCAAGA	GCCACCACCGTGGGAACTGT
TGM1	NM_001003079	CGGGTGGCAAGTGGTAGACGC	TGGATTCCACAGAGCAGGGGCC

[a]Housekeeping Gene

Files S2-S4

Available for download as Excel files at http://www.g3journal.org/lookup/suppl/doi:10.1534/g3.114.013003/-/DC1

File S2: Excel spread sheet showing the data after statistical unpaired analysis; the feature number is the sample number in the array, following rows show the different type of gene Ids, thereafter the coefficient of differential expression, the t-value, the p-value, the adjusted p-value and the F values. The last row indicates if the expression is higher (1) or lower than the mean value (-1), the colored rows show human genes that matched with the canine ones to identify more functions for the non annotated canine genes.

File S3: Excel spread sheet with the results of the DAVID analysis of the gene sets 1 and 2; the Annotation Clusters with the highest enrichment score are shown first, the terms give the functional group in wich the genes are classified; in the row GENES the human orthologous gene IDs are listed.

File S4: The results of the DAVID analysis of genes that were classified in Clusters 1- 4 in the SOTA analysis; the annotation clusters with the highes enrichment score are shown first, the row "Term" shows the functional group where the genes were classified in, in the row F "Genes" gene Ids of the genes matching this category are listed.
