# Supplementary material for: Gene Expression in the Skin of Dogs Sensitized to the House Dust Mite Dermatophagoides farinae
Source: G3 (Bethesda). 2014 Aug 5;4(10):1787–95. doi: 10.1534/g3.114.013003 (PMC4199687; doi:10.1534/g3.114.013003)
Supplement: Supporting Information [file supp_g3.114.013003_TableS1.pdf]

Table S1 Design of specimen collection; each biopsy was cut into three pieces - one of these was used for histological analysis, the other two parts were used for microarray analysis

day	6 sensitized dogs	6 non sensitized dogs
4 days before patch test	clipping the left lateral thorax	
before patch test (0h)	1 biopsy (non treated skin) / dog	
patch test	patch test with allergen*/saline	patch test with allergen*/saline
+6 h	1 biopsy of each treatment-area (allergen and saline = 2 biopsies / dog)	1 biopsy of each treatment-area (allergen and saline = 2 biopsies / dog)
+24 h	1 biopsy of each treatment area (allergen and saline = 2 biopsies / dog)	1 biopsy of each treatment area (allergen and saline = 2 biopsies / dog)
number of taken biopsies	30	30
