# Supplementary material for: Gene Expression in the Skin of Dogs Sensitized to the House Dust Mite Dermatophagoides farinae
Source: G3 (Bethesda). 2014 Aug 5;4(10):1787–95. doi: 10.1534/g3.114.013003 (PMC4199687; doi:10.1534/g3.114.013003)
Supplement: Supporting Information [file supp_g3.114.013003_TableS2.pdf]

Table S2 Sequences of the oligonucleotides used as primers for qPCR

GeneID	Accession Number	Forward Primer	Reverse Primer
[a]RPL13A	XM_533620	CTGCCCCACAAGACCAAG	GGGATCCCATCAAACACCT
[a]LOC479750	XM_536878	GCAGGAAGGGATTCTCCAG	GGTCCAGTAAGAAATCTTCCATAA
[a]UBB	XM_858590	CGCGGCTGGCATTGTTAGCTG	GCATACCACCCCTCAGGCGC
ALOXE3	XM_546605	GCACCTGTGGCCAGAGCCCTA	CAGCTCCGTGGAGCAGCACAC
CCL8	NM_001005255	TGCCAGCTTCAGCACCTTTGTCC	TGGGGTCAGCACAGATCTCCCTG
CLEC7A	XM_849050	TCAAGGCATCACTGGGCGACCT	TCACAGCAATGGGACGCCAACG
DMKN	XM_533694	CTGGGGGTCAGGGCTTCGGA	TCCTCCACTGGACCCGTGCC
DSG1	NM_001002939	CCCAGCAGCGATGAACTGGCA	TCACGACAGGCTGCAGCGAA
DSP	XM_545329	TTCTGCAGGAGGAGGGCACCC	GTGGCCTTCAGGCGAGTGAGC
FCGR3A	XM_536141	TGGCTGCACAATGGGAGACCCAT	GAGGGACCTGGAGCAAGAGCCA
FLG2	XM_540329	TGGCACACTGAGCAAGGATGAGC	GCTGAGAACCTTGTTGCAGGCCA
IL13RA2	NM_001003075	TGGAAACCTGGCATGGGTGTCC	TCTGCTGAATGGTCCAAGCCCTCA
IL18BP	NM_001048018	GCCTCTCCTGGCTCCGGACA	CCGACCTGGGAGGTGCTCGA
IL33	NM_001003180	TTTGCTGCATGCCAACAACGAGG	AGGAAGAAGGCCTGGTCTGGCAA
KPRP	ENSCAFT00000 020644	AAGTCCCTGTCCACGTCCTGCT	CGTGGCTCGGGAAATTCACGCT
LOC476953	XM_534152	CAGCACCAGCATCCCAGCTCC	CCCGTGCTTGGGATGGCACT
OCLN	NM_001003195	GCTCTGGGATCCTGCTCGTCCT	CGTGCATGTCCCCACCGTACAC

PPARA	NM_001003093	AAAGCCCGGGTCATCCTCGC	GCGCACCTCTGCCTCCTTGTT
SOCS3	NM_001031631	TGAACGCAGTGTGCAAGCTGC	AGCGTGAAGAAGTGGCGCTGG
SPINK5	NM_001025397	GCGTGGCCCAGATGGCAAGA	GCCACCACCGTGGGAACTGT
TGM1	NM_001003079	CGGGTGGCAAGTGGTAGACGC	TGGATTCCACAGAGCAGGGGCC

[a]Housekeeping Gene
